# Defining indicators for disease burden, health outcomes, policies and barriers and facilitators to health services for migrant populations in the Middle East and North African region: a protocol for a suite of systematic reviews

**DOI:** 10.1136/bmjopen-2023-083813

**Published:** 2024-07-12

**Authors:** Farah Seedat, Stella Evangelidou, Moudrick Abdellatifi, Oumnia Bouaddi, Alba Cuxart-Graell, Hassan Edries, Eman Elafef, Taha Maatoug, Anissa Ouahchi, Liv Mathilde Pampiri, Anna Deal, Sara Arias, Adel Abdelkhalek, Ahmed Hamed Arisha, Bouchra Assarag, Ibrahim Ahmed Bani, Aasmaa Chaoui, Wafa Chemao-Elfihri, Kenza Hassouni, Mahmoud Hilali, Mohamed Khalis, Wejdene Mansour, Ali Mtiraoui, Kolitha Wickramage, Dominik Zenner, Ana Requena-Mendez, Sally Hargreaves, MENA Migrant Health Working Group, MENA Migrant Health Working Group, Adel Abdelkhalek, Asad Adam, Adnene Ben Haj Aissa, Charles Agyemang, Salma Altyib, Ali Ardalan, Hanen Ben Belgacem, Imane Belkhammar, Thomas Calvot, Nuria Casamitjana, Luciana Ceretti, Nelly Chavassieux, Hassan Chrifi, Mohamed Douagi, Algdail Elnil, Gonzalo Fanjul, Fouad M Fouad, Ahmed Hamed, Chiaki Ito, Abdedayem Khelifi, Lora Makhlouf, Maissa Mokni, Davide Olchini, Tarik Oufkir, Nasong Park, Giuseppe Raffa, Wafa Saidi, Sandra Santafé, Alice Sironi, Fatma Temimi, Zeineb Turki

**Affiliations:** 1 St George's University of London Institute for Infection and Immunity, London, UK; 2 Barcelona Institute for Global Health, Barcelona, Catalunya, Spain; 3 Université Mohammed VI des Sciences et de la Santé, Casablanca, Casablanca-Settat, Morocco; 4 University of Gezira, Wad Madani, Sudan; 5 Badr University in Cairo, Cairo, Egypt; 6 University of Sousse, Sousse, Tunisia; 7 London School of Hygiene & Tropical Medicine, London, UK; 8 Ecole Nationale de Santé Publique, Rabat, Morocco; 9 Ajman University, Ajman, UAE; 10 Ministère de la Santé, Rabat, Morocco; 11 Higher Institute of Nursing Professions and Health Techniques, Ministry of Health and Social Protection, Rabat, Morocco; 12 International Organization for Migration, Berlin, Germany; 13 Queen Mary University of London, London, UK; 14 Karolinska Institute, Stockholm, Sweden

**Keywords:** EPIDEMIOLOGY, Public health, PUBLIC HEALTH, Systematic Review, MENTAL HEALTH, Health policy

## Abstract

**Abstract:**

**Introduction:**

The Middle East and North African (MENA) region is characterised by high and complex migration flows, yet little is known about the health of migrant populations, their levels of underimmunisation and access to healthcare provision. Data are needed to support regional elimination and control targets for key diseases and the design and delivery of programmes to improve health outcomes in these groups. This protocol describes a suite of seven systematic reviews that aim to identify, appraise and synthesise the available evidence on the burden and health outcomes, policies and access (barriers and facilitators) related to these mobile populations in the region.

**Methods:**

Seven systematic reviews will cover three questions to explore the: (1) burden and health outcomes, (2) policies and (3) healthcare barriers and facilitators for the following seven disease areas in migrants in the MENA region: tuberculosis, HIV and hepatitis B and C, malaria and neglected tropical diseases, diabetes, mental health, maternal and neonatal health, and vaccine-preventable diseases. We will search electronic databases for studies in any language (year 2000–2023), reference-check relevant publications and cross-check included studies with experts. We will search for grey literature by hand searching key databases and websites (including regional organisations and MoH websites) for country-specific guidelines and talking to our network of experts for local and regional reports and key datasets. We will assess the studies and policies for their quality using appropriate tools. We will meta-analyse the data by disease outcome if they are of sufficient volume and similarity. Where meta-analysis is not possible and where data are on policy or access, we will narratively synthesise the evidence using summary tables, figures and text.

**Dissemination:**

We anticipate disseminating the findings through peer-reviewed publications, conferences and other formats relevant to all stakeholders. We are following Preferred Reporting Items for Systematic Reviews and Meta-Analyses guidelines and protocols will be registered on International Prospective Register of Systematic Reviews.

**STRENGTHS AND LIMITATIONS OF THIS STUDY**

- Our systematic reviews use explicit, transparent and recommended methodology.
- We will apply comprehensive search strategies with no language restrictions and extensive grey literature searching online and through expert government and non-government support.
- We have an international team with multidisciplinary expertise on migrant health, diseases, policy-making and methodology.
- Data prior to the year 2000 will be excluded.
- Meta-analysis may not be possible due to heterogeneity, and we anticipate that most of the data will come from grey literature.

## Introduction

 The Middle East and North African (MENA) region is marked by political and economic instability, extended conflict and high and complex migrant flows. Several countries in this region are points of origin, transit and destination for a varied group of migrants. More than 40 million migrants reside in the region,^1^ including 12.6 million internally displaced people (IDPs), 2.4 million refugees, 251 800 asylum seekers and 370 300 stateless persons at the end of 2022.^2^ Mass displacement in Sudan and Palestine have intensified the situation, with reports of over 7 million people becoming newly displaced in Sudan (over 1.38 million fleeing to neighbouring countries) while 1.9 million have been internally displaced in Gaza.^3 4^

Migrants are a heterogeneous group; while some are resilient throughout the migration cycle, have sufficient access to services in host countries and good health outcomes, others in the region live and work in precarious conditions, and may face individual-level and system-level barriers in accessing healthcare.^5^,^7^ Efforts have been made to include migrants in national health programmes and enable them access to affordable, acceptable, culturally sensitive and good quality healthcare. For example, Morocco has multisectoral programmes through a national immigration and asylum strategy since 2013 to improve the health and well-being of all migrants, which includes the right of access to free or low-cost essential healthcare under the same conditions as Moroccans.^8 9^ However, migrants may still face economic barriers such as the cost of medication and complimentary tests, which may not be covered, as well as language and cultural barriers, a fear of deportation, racism and discrimination.^6 10^ Non-governmental organisations (NGOs) and United Nations (UN) agencies attempt to close the gap in healthcare provision, but these services often remain insufficient.^7 11^ This context can leave some migrant groups disproportionately affected by various health conditions, with poor morbidity and mortality outcomes.^12^

Globally, it is reported that certain migrant categories may encounter infectious diseases such as tuberculosis (TB) and HIV/AIDS along their journey and in their host countries, and they may be at risk of malaria and neglected tropical diseases (NTDs) for which health professionals may be unfamiliar. There is also evidence that they are disproportionately affected by the burden and consequences of COVID-19.^13^ Likewise, some migrants, especially refugees and asylum seekers, can be at risk of non-communicable diseases (NCDs), which are often diagnosed late and can be causes of premature mortality.^7 12 13^ In addition, depending on their lived experiences during the migratory trajectory and the contextual factors in the host country, some migrants may suffer adverse mental health outcomes.^13^ Refugee and migrant women tend to have less access to maternal and child health services and are at a higher risk of negative outcomes during pregnancy and delivery than women in host populations.^13^ Migrants are also considered to be an underimmunised group, missing vaccines, doses and boosters as children and adolescents because of their mobility, with WHO calling for greater emphasis to be placed on vaccination across the life course in marginalised groups.^14 15^

There is a paucity of data mapping the burden of diseases and underimmunisation in migrants in the MENA region^13^; however, some small studies in some countries suggest that migrants may be at higher risk for some diseases. For example, in Lebanon, migrants are considered a vulnerable group for HIV as the political and economic situation has led to an increase in high-risk behaviours,^16^ while cross-border mobile populations in the ports of the Red Sea and adjacent transport corridors are found to be at a higher risk of HIV transmission in the Horn of Africa and Arabian Peninsula.^17^ Similarly, high rates of hepatitis B and C have been found in newly arriving sub-Saharan migrants in Libya (23.4% and 31.2%, respectively),^18^ and migration has been identified as partly explaining the slowing of the decline in TB and the reintroduction of malaria in the region,^19 20^ as well as outbreaks of neglected tropical diseases such as leishmaniasis and cysticercosis/taeniasis in Lebanon.^21^

The evidence on NCDs is similar. A scoping review in 2019 on Syrian refugees found only two studies that were investigating prevalence—one in Lebanon and one in Jordan.^22^ The studies found that almost one in two households had a member with an NCD; the two most common NCDs were hypertension and type 2 diabetes mellitus. In Lebanon, the proportion of NCDs was higher in the refugees than the host community (60.2% vs 50.4%, respectively). A retrospective study in Qatar similarly found that diabetes and hypertension were higher in migrants who had just arrived (<6 months) compared with longer durations.^23^ There is also some evidence of a high burden of psychiatric disorders, such as generalised anxiety disorder and post-traumatic stress disorder in Lebanon, Sudan and Egypt among refugees and IDPs with premigration exposure to armed conflict.^24^ The Gulf Cooperation Council reported high rates of psychosis and suicide among migrant domestic workers coming from Indian Subcontinent and South East Asia.^25^ With respect to maternal and neonatal conditions, a study in Lebanon found that the odds of very preterm birth and other serious antenatal complications were higher for most migrant women compared with host women.^26^

There is equally little information on the policies and interventions (eg, screening, diagnostic, treatment or vaccination services) available to migrants in the MENA region. Indeed, when exploring the NCD policies for urban refugees, a scoping review in 2020 concluded that there is a scarcity of research on national policy, the prevention of NCDs and the perspective of refugees.^27^ Likewise, there are little comprehensive data about infectious disease policies among migrants in the MENA region. Two Sudanese studies reported a low vaccination coverage among migrant and internally displaced children against major vaccine-preventable diseases (VPDs).^28 29^ In countries such as Egypt, Iraq, Jordan, Lebanon, Morocco and Tunisia, migrants are included in vaccination policy irrespective of their residency status. However, in some countries, some groups of migrants often cannot access sufficient vaccinations, and NGOs are left to fill this gap, especially for irregular migrants.^14^

**Figure 1:**
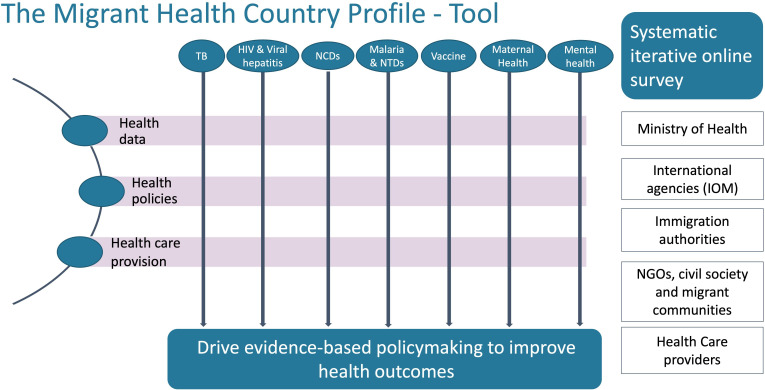
Schematic illustration of the Migrant Health Country Profile Tool (MHCP-t). NTD, neglected tropical diseases; TB, tuberculosis.

The lack of comprehensive data on the burden of diseases among migrants in the MENA region makes it difficult to respond to the unmet healthcare needs of the migrant population.^1 30^ Likewise, the extent to which migrants are included in national policies and the barriers they may face could undermine the goals to achieve the legal and human right of universal health coverage, ensure health security for all, and eliminate some of these diseases. Efforts are underway to make integrated migrant health information systems a reality in the MENA region. Since 2015, the International Organization for Migration (IOM) has steered projects fostering health and protection to vulnerable migrants transiting through Morocco, Tunisia, Egypt, Libya, Yemen and more recently, Sudan.^31^ The current phase aims to steer the development of a migrant health country profile tool (MHCP-t) for the region, an innovative digital mechanism to source country-level data on health indicators, health policies and healthcare access across multiple communicable and NCD areas. The MHCP-t will enable a systematic assessment of disease impact on migrants and identify country-level hotspots and gaps in prevention efforts by integrating information from routine health data, registries, surveillance programmes, humanitarian stakeholders and civil society, on health aspects of migrant populations (see figure 1).

A preliminary step in the development of the MHCP-t is to map out the state of the evidence on migrant health data and policies in the MENA region and to inform the development of key indicators for disease burden and health outcomes, policies and access for all relevant disease areas of the MHCP-t. We will also conduct qualitative field studies with migrants, community leaders and healthcare professionals in Morocco, Tunisia and Egypt to further inform the key indicators. The resulting key indicators will be reviewed by national task groups, brought together by the ministries of health, and international experts. The final list of indicators will be developed into the first version of the tool, which will be piloted within the countries using a mixed-methods process evaluation.

To date, there have been no attempts to systematically synthesise the disease, policy or access indicators in the literature about key diseases among migrant populations in the MENA region. This suite of systematic reviews will aim to systematically identify, appraise and synthesise the empirical evidence on key diseases among migrant populations in the MENA.

### Research questions and objectives

There are three overarching research questions that will be addressed in seven systematic reviews on seven disease areas to comprehensively map the state of the evidence of migrant health in the MENA region: (1) TB, (2) HIV and hepatitis B and C, (3) malaria and NTDs, (4) diabetes, (5) mental health, (6) maternal and neonatal health and (7) VPDs.

1. What data are available on the disease indicators related to each disease area in migrant populations in the MENA region?

Objectives:

- Synthesise and appraise data on the burden (eg, prevalence or incidence) of TB, HIV, hepatitis B and C, malaria, NTDs, diabetes, mental health, maternal and neonatal health conditions, and VPDs in migrant populations in the MENA region.
- Synthesise and appraise data on intervention outcomes (eg, uptake and coverage of vaccination, screening or treatment, treatment success) related to TB, HIV, hepatitis B and C, malaria, NTDs, diabetes, mental health, maternal and neonatal health, and VPDs in migrant populations in the MENA region.
- Synthesise and appraise data on the intermediate (eg, symptoms, severity, or prognosis/long-term morbidity of disease) and final (eg, mortality, quality of life) health outcomes of TB, HIV, hepatitis B and C, malaria, NTDs, diabetes, mental health, maternal and neonatal health conditions, and VPDs in migrant populations in the MENA region.

2. What is the policy response for each disease area related to migrant populations in the MENA region?

Objective:

- Synthesise and appraise the prevention and/or treatment policies for TB, HIV, hepatitis B and C, malaria, NTDs, diabetes, mental health, maternal and neonatal health and VPDs in migrant populations in the MENA region.

3. What are the barriers and facilitators in accessing health services for each disease area for migrant populations in the MENA region?

Objective:

- Synthesise and appraise the evidence on the barriers and facilitators for accessing prevention and/or treatment services for TB, HIV, hepatitis B and C, malaria, NTDs, diabetes, mental health, maternal and neonatal health and VPDs in migrant populations in the MENA region.

We will disaggregate by, and investigate potential sources of heterogeneity for, country of study, study period, country of origin, migrant type (ie, labour, asylum seekers, refugee), age and sex, where feasible for all objectives, and cross-compare findings across countries in the MENA region.

## Methods and analysis

The seven systematic reviews will be reported according to recommendations from the Preferred Reporting Items for Systematic Review and Meta-Analysis Protocols (PRISMA-P) 2020 statement.^32^ We will register the systematic reviews protocols at the International Prospective Register of Systematic Reviews.

### Search strategies

#### Electronic database searches

We will search the following electronic databases to identify peer-reviewed literature: Medline (Ovid); Embase (Embase.com); Web of Science (Clarivate), CINAHL (Ebsco), Index Medicus for the Eastern Mediterranean Region (globalindexmedicus.net) and Qscience (qscience.com). Scoping searches have been undertaken to inform the development of the search strategies. An iterative procedure was used, with input from all authors including an information scientist, recommended search filters, and previous reviews.^33^,^37^ The searches are separated for each disease area. They combine three sets of search terms using both free text words in title, abstract or keyword heading word, and MeSH terms through boolean operators OR within each set and then AND to combine the sets. The first set is made up of search terms for migrants, the second set is made up of search terms for each disease area and the third set is made up of search terms for the MENA region. The search strategies are limited to humans and restricted to the year 2000 as migratory flows and disease burden change over time, and we are interested in recent migration flows and recent policies. There is no restriction on language. A copy of the overarching search strategy used in the major databases is provided in table 1, and the strategies specific to each disease area (ie, terms for diseases) are presented in the online supplemental material.

**Table 1:** Overarching search strategy for all reviews

1	Migrant	(alien* or asile or asylum* or (border* adj2 cross*) or (countr* adj3 origin*) or diaspora or displace? Or displacement* or emigrant* or emigration or expat? Or expatriate? Or foreigner* or foreign-born* or foreign background* or foreign population* or immigrant* or immigration or migrant* or migration or naturalized citizen* or new* arriv* or newcomer* or new-comer* or nomad* or non-citizen* or nonnative* or non-native* or nonnational or non-national or nonresident or non-resident* or resettlement* or re-settlement* or refugee* or settler* or squatter* or undocumented worker*).ti,ab,kf.Or exp Human Migration/ or exp “Emigrants and Immigrants”/ or “Transients and Migrants”/ or Refugees/ or Refugee Camps/
2	Disease	Separate for each strategy (see online supplemental material)
3	Countries	(Abu Dhabi or Ajman or Algeri* or Arab* or Bahrain* or Bahreiin* or Dubai or Egypt* or Emirat* or Fujairah or Gaza* or Golf* or Gulf* or Ifriqiya* or Irak* or Iraq* or Jorda* or Jumhuuriiya* or Koweit* or Kuwait* or Kuwayt* or Leban* or Liban* or Liby* or Lubnan* or Maghr* or Maroc* or Maser* or Masr or Misr or MENA or Middle East* or Morocc* or North* Afric* or Oman* or Palestin* or Qatar* or Saudi* or Sharjah or Soudan* or Sudan* or Syria* or Syuri* or Tunis* or Uman* or Umm Al-Quwain or West Bank or Yemen*).ti,ab,kf.Exp middle east/ Exp Africa, Northern/exp Middle East / or exp Africa, Northern/
4	Combine for indicators	1 AND 2 AND 3
5	Limit to humans	(animals not humans).sh.4 not 5
	Limit to 2000	Limit 5 to yr=”2000-Current”

#### Grey literature searches

We will search various sources to identify literature that has not been published in peer-reviewed journals. First, we will search the reference lists of the final list of included studies at full text as well as systematic reviews that meet our criteria that we have sourced from searches of the electronic databases. Second, we will search international and regional websites related to migration and health, such as the IOM, WHO, UNHCR, Red Cross and other relevant UN bodies, Médecins San Frontières (MSF) and other key regional NGOs, and national websites for each country in the MENA region, particularly ministries of health, for guidelines, policies, reports and unpublished datasets. We will use snowballing methodology and search any relevant websites we find from these initial websites, as necessary. We will report every organisation we search in the write up of the review. Once we have a full list of included papers and websites, we will share this with a panel of country-specific and regional experts to review and identify any further data sources that have not been captured.

### Study screening and selection

**Figure 2:**
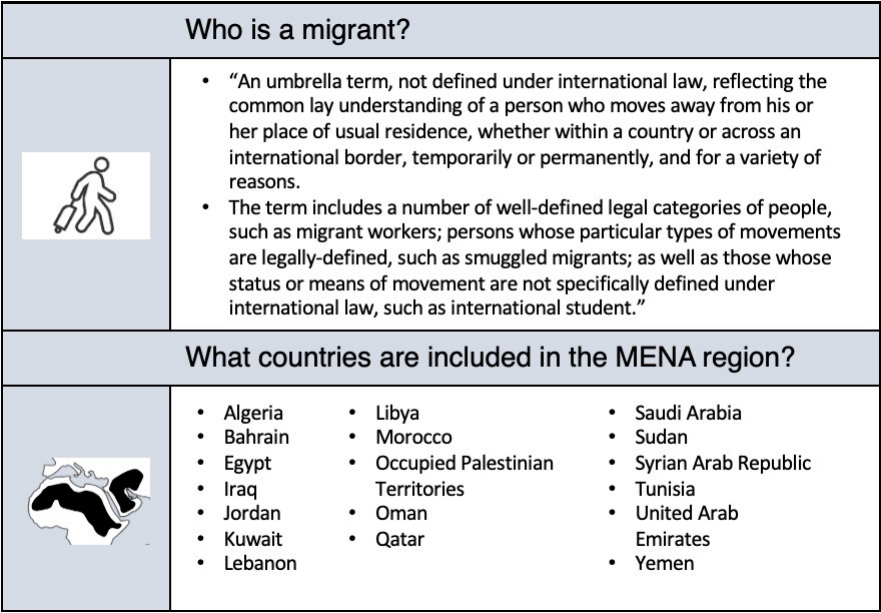
Definitions used in this project, adopted from the International Organization for Migration. References: International Organization for Migration. IOM definition of ‘Migrant’ Switzerland: International Organization of Migration (available from: https://www.iom.int/about-migration, accessed 4 April 2023). International Organization for Migration. IOM Middle East and North Africa regional strategy 2017–2020. Cairo 2017.

The eligibility criteria by disease area are summarised separately in the table 2 for disease indicators (question 1) and table 3 for policy (question 2) and access (question 3). For disease indicators (question 1), we will include papers that are on the burden (eg, prevalence or incidence), or intervention (eg, uptake, coverage, or completion of interventions such as screening or treatment) or intermediate and final (eg, symptoms, severity, long-term morbidity, mortality, quality of life) health outcomes for TB, HIV, hepatitis B and C, malaria, NTDs, diabetes, mental health, maternal and neonatal health conditions, and VPDs in migrant populations in the MENA region. For policy-related data (question 2), we will include papers that contain a description of the policies themselves, and for access data (question 3) we will include papers that are on determinants of any usage/underusage, facilitators or barriers in accessing health services for the disease areas in migrant populations in the MENA region. Definitions for migrant and the MENA region are described in figure 2.

**Table 2:** Study eligibility criteria for disease indicators (question 1)

Disease	Participants	Intervention/exposure	Comparators	Outcomes	Study design	Publication andlanguage	Exclusions
Overall	Migrants in living in the MENA region.	None or any as reported by the authors	No intervention, counterfactual or control group (eg, host population), different migrant subgroups, or alternative intervention, as reported by authors	Three categories of outcomes: (1) burden (eg, incidence or prevalence); (2) intervention outcomes (eg, uptake, coverage, completion or success of interventions); (3) intermediate (eg, symptoms or severity of disease, long-term morbidity) and final health outcomes	Cohort studies, cross-sectional studies, case-control studies, trial studies (data will only be extracted from baseline characteristics or the control group as appropriate), systematic or narrative reviews (only to find relevant included primary studies), data, and reports from national/regional surveillance systems	Full-text reports in any language.If an insufficient number of full-text reports are found, we will include abstracts, editorials, letters, consensus statements, or opinions.No language restrictions.	Studies on diagnostic accuracy or cost-effectiveness of interventions. Data on the clinical effectiveness will also not be included; however, relevant baseline or control group data from these studies may be included.Case series and case reports.Abstracts, editorials, letters, books, consensus statements, or opinions if there are sufficient full texts.
TB		For example, latent, and active TB screening, diagnosis, and antibacterial treatment, and so on.		Burden (eg, incidence or prevalence of active, latent or MDR TB, etc); (2) intervention outcomes (eg, uptake or coverage of active and latent TB screening, diagnosis, TB chemoprophylaxis, or treatment for active TB, treatment completion, cases lost to follow-up, microbiologic cure/successfully treated cases, etc); (3) intermediate and final health outcomes (eg, cases with drug resistance, comorbidities, extrapulmonary TB, TB meningitis, mortality, quality of life, quality-adjusted life years, disability-adjusted life years, etc, for patients with TB)			
HIVhepatitis B and C		For example, antiretroviral therapy, health education, condom prevention, counselling and so on.		Burden (eg, incidence, prevalence, etc, of HIV or hep B and C); (2) intervention outcomes (eg, uptake or coverage of HIV or hep B and C screening and testing, knowledge of results, uptake or coverage of HIV or hep B and C treatment, cases lost to follow-up, treatment success, etc); (3) intermediate and final health outcomes (eg, viral load, comorbidities, disease stage, mortality, quality of life, quality-adjusted life years, disability-adjusted life years, etc, for patients with HIV or hepatitis B and C)			
MalariaNTDs		For example, chemoprophylaxis, health education, vector control, and so on.		Burden (eg, incidence, prevalence, etc, of malaria or NTDs); (2) intervention outcomes (eg, uptake and coverage of: malaria prevention and treatment-related messages, use of an insecticide-treated net, spraying of IRS, first-line antimalarial treatment, prevention, treatment and care for any NTDs, cases loss to follow-up, treatment success, etc); (3) intermediate and final health outcomes (eg, blindness, anaemia, malnutrition, neurological abnormalities, haemoglobin levels, mortality, quality of life, quality-adjusted life years, disability-adjusted life years, etc, for patients with malaria or NTDs)			
VPDs		Vaccination		Burden (eg, incidence, prevalence, etc, of VPDs); (2) intervention outcomes (eg, uptake or coverage of vaccination); (3) intermediate and final health outcomes (eg, severity, long-term illness, mortality, quality of life, disability-adjusted life years, quality-adjusted life years, etc, for patients with VPDs)			
MNH		For example, antenatal care, postnatal care, and so on.		Burden (eg, incidence, prevalence, etc, of preeclampsia, postpartum haemorrhage, low birth weight, preterm birth, etc); (2) intervention outcomes (eg, uptake or coverage of antenatal care attendance, skilled delivery attendance, antenatal screening, newborn screening, or treatments, cases lost to follow-up, treatment success, etc); (3) intermediate and final health outcomes (eg, breathing and feeding difficulties, vision or hearing problems, developmental delays, cerebral palsy, maternal mortality, neonatal mortality, stillbirth, quality of life, disability-adjusted life years, quality-adjusted life years, etc, for mothers or newborns with health conditions)			
Diabetes		For example, educational support, insulin injections, and so on.		Burden (eg, incidence, prevalence, etc. of type I and II diabetes); (2) Intervention outcomes (eg, uptake and coverage of diabetes prevention programmes, screening, or treatment for diabetes, loss to follow-up, etc); (3) Intermediate and final health outcomes (eg, retinopathy, heart attacks, stroke, neuropathy, mortality, quality of life, disability-adjusted life years, quality-adjusted life years, etc, for patients with diabetes)			
Mental health		For example, psychiatric care, community mental health programmes, MHPSS response, and so on.		Burden (eg, incidence, prevalence, etc, of any mental health conditions); (2) intervention outcomes (eg, uptake and coverage of primary prevention, screening, talking therapy, psychiatric treatment, loss to follow-up, treatment success, etc); (3) intermediate or final health outcomes (eg, stage of condition, functioning, development capabilities, mortality, quality of life, disability-adjusted life years, quality-adjusted life years, etc, for patients with mental health conditions)			

HephepatitisHep, hepatitis; HIV, HIV immunodeficiency virus; HIVhuman immunodeficiency virusIRSIndoor residual sprayingIRS, Indoor residual spraying, MENA=Middle East and North Africa, MDR=multi drug resistant, MNH=maternal and neonatal health, MHPSS=Mental Health and Psychosocial Support NTD=Neglected tropical diseases, TB=Tuberculosis, VPD=vaccine-preventable diseaseMDRmultidrug resistantMENAMiddle East and North AfricaMHPSSMental Health and Psychosocial SupportMNHmaternal and neonatal healthNTDneglected tropical diseasesTBtuberculosisVPDvaccine-preventable disease

**Table 3:** Study eligibility criteria for policy and access indicators (questions 2 and 3)

Disease	Participants	Intervention/exposure	Comparators	Outcomes	Study design	Publication andlanguage	Exclusions
Question 2. Policy indicators							
TB	Migrants in the MENA region	Policies for health services related to TB	No intervention, counterfactual or control group (eg, host population), different migrant subgroups, or alternative intervention, as reported by authors	Description of the policy itself	Any study design, guideline, reports, policy or programme information.	Full-text reports in any language.No language restrictions.	Studies on diagnostic accuracy, clinical effectiveness or cost-effectiveness of interventionsCase series and case reports.Abstracts, editorials, letters, books or opinions if there are sufficient full texts.
HIVHep B and C		Policies for health services related to HIV and hep B and C					
Malaria NTDs		Policies for health services related to malaria and NTDs					
VPDs		Policies related to vaccination					
MNH		Policies for health services related to maternal and neonatal health					
Diabetes		Policies for health services related to diabetes					
Mental health		Policies for health services related to mental health					
Question 3. Access indicators							
TB	Migrants in the MENA region	Health services related to TB	No intervention, counterfactual or control group (eg, host population), different migrant subgroups, or alternative intervention, as reported by authors	BarriersFacilitatorsDeterminants of any usage/underusage	Cohort studies, cross-sectional studies, case-control studies, descriptive studies, qualitative studies, systematic or narrative reviews (only to find relevant included primary studies), reports from national/regional programmes	Full-text reports in any language.No language restrictions.	Studies on diagnostic accuracy, clinical effectiveness or cost-effectiveness of interventionsCase series and case reports.Abstracts, editorials, letters, books or opinions if there are sufficient full texts.
HIVHep B and C		Health services related to HIV and hep B and C					
Malaria NTDs		Health services related to malaria and NTDs					
VPDs		Vaccination					
MNH		Health services related to maternal and neonatal health					
Diabetes		Health services related to diabetes					
Mental health		Health services related to mental health					

Hep, hepatitis; HIV, immunodeficiency virus; MENA, Middle East and North Africa; MNH, maternal and neonatal health; NTD, neglected tropical diseases; TB, tuberculosis; VPD, vaccine-preventable disease

For disease areas that concern multiple diseases, we have prioritised those that are of greater concern in the MENA region. We are including the following VPDs: cholera, COVID-19, dengue, diphtheria, hepatitis A, hepatitis B; haemophilus influenza type b, human papillomavirus, influenza, measles, meningococcal diseases, mumps, pertussis, pneumococcal disease, poliomyelitis, rabies, rubella, rotavirus, tetanus, TB, typhoid and varicella. For NTDs, we will include buruli ulcer, dengue and chikungunya, dracunculiasis (Guinea-worm disease), echinococcosis, human African trypanosomiasis (sleeping sickness), leishmaniasis, leprosy (Hansen’s disease), lymphatic filariasis, mycetoma, and other deep mycoses, onchocerciasis (river blindness), rabies, scabies and other ectoparasitoses, schistosomiasis, soil-transmitted helminthiases, snakebite envenoming, taeniasis/cysticercosis, trachoma, and yaws and other endemic treponematose. We are including any mental health condition, and for NCDs, we have chosen diabetes for this first stage. In the next stage, we will do a wider systematic review on NCDs in migrant populations in the MENA region, including hypertension, cardiovascular diseases, obesity and chronic kidney disease.

With respect to interventions, for disease indicators, we will include studies in which any intervention or exposure is reported, or studies where no intervention is reported if the study is about prevalence or incidence alone. For policy and access data, we will include any health interventions relevant for the disease area. For all questions, we will include studies that have no comparator or any type of comparator (ie, a counterfactual group, control group, reference group for an exposure, different migrant subgroup, or an alternative intervention). For publication type, we will include full texts of observational studies, data from national and regional surveillance systems, and trials for the question on disease indicators, although for trials, we will only extract data from the control group or from baseline characteristics, as appropriate. For access data, we will include full-text papers from observational or qualitative studies, or programme reports. For both questions 1 and 3, we will include systematic and narrative reviews only to search for relevant included studies from these papers. For policy data, we will include full-text papers from any study design, guideline or policy and programme documentation. We will exclude studies on diagnostic accuracy, cost effectiveness of interventions, case series and case reports, and exclude abstracts, editorials, books or opinions, if there are a sufficient number of other publications. We will have no language exclusions as within our coauthors we have speakers of the MENA region (Arabic, French and English) as well as Spanish; if we find an article in another language, we will use a professional translator or an online automated translation service, depending on the cost and budget.

Identified references will be downloaded to bibliographic management software, Rayyan, and deduplicated. Two reviewers will independently screen the titles and abstracts of all identified records (screening level I). Full-text reports of all potentially relevant records identified at screening level I will be obtained and assessed independently by two reviewers using the same study eligibility criteria (screening level II). Any disagreements over inclusion/exclusion at screening level I and II will be resolved by discussion between the two reviewers, with the involvement of a third reviewer if necessary. We will document the study flow and reasons for exclusion of full-text papers in a PRISMA study flow diagram (see online supplemental material).

### Data extraction

Two reviewers will independently extract relevant data using an a priori defined extraction sheet that will be piloted and refined before implementation. Data extracted will be cross-checked and any disagreements will be resolved by discussion, with the involvement of a third reviewer if necessary.

Data extracted for disease indicators (question 1) will include study (eg, author, country, publication year, design, setting, sample size, follow-up duration), participant (eg, type of migrant and definition used, country of origin, time in the country, sociodemographic characteristics, living situation (camp setting, community), study eligibility criteria), health or disease indicators including their definitions (eg, description of the intervention if it is about testing or education or treatment), the outcome measures (eg, frequency, percentages, 95% CIs, etc), results reported for each outcome, and any adjustment conducted for confounding of the outcomes. From each study that has adjusted and unadjusted analyses, we will prioritise the adjusted analysis if data allow. Any missing statistical parameters of importance and variability measures (eg, 95% CIs) will be estimated, if data permit. All calculated or derived data will be denoted as ‘calculated’ and will be incorporated in the extraction sheets.

Data extracted on policies (question 2) will include publication (eg, author, country, publication year, publication type), population covered by policy (eg, type of migrant and definition used, sociodemographic characteristics), summary of interventions covered by policy (and comparators (if appropriate), setting (eg, camp, community), summary of policy, level of authority ((national, regional, local) implementation (legal, recommendation, guideline) and methods used to inform policy. Data extracted on access (question 3) will include study (eg, author, country, publication year, design, setting, sample size, follow-up duration), participant (eg, type of migrant and definition used, country of origin, time in the country, sociodemographic characteristics, living situation (camp setting, community), study eligibility criteria), interventions in the study (if necessary), list of barriers, facilitators or determinants of usage/under-usage, outcome measures and results (eg, frequency, percentages, 95% CIs, etc) where appropriate, and any adjustment conducted for confounding of the outcomes. Any missing statistical parameters of importance and variability measures (eg, 95% CIs) will be estimated, if data permit. All calculated or derived data will be denoted as ‘calculated’ and will be incorporated in the extraction sheets.

### Risk of bias

Two independent authors will appraise the risk of bias for each included paper. For peer-reviewed literature, we will use the appropriate Joanna Briggs Institute tool for each study design and for grey literature records, we will use the Authority, Accuracy, Coverage, Objectivity, Date and Significance (AACODS) checklist.^38 39^ We will assess the quality of the policies using the most appropriate AGREE tool—AGREE II or AGREE HS.^40^ The quality appraisals will be cross-checked and any disagreements will be resolved by a consensus-based discussion, with the involvement of a third reviewer if necessary. The individual item-specific quality assessment ratings for each study will be tabulated. Records will not be excluded based on quality assessment, but the appraisal will contribute to the synthesis and the discussion.

### Data synthesis and analysis

#### Disease indicators (question 1)

If the studies are sufficiently similar, we will combine the data using a proportional meta-analysis via a random effects model due to the anticipated heterogeneity that may result from the differences in methodology and study settings.^41^ There will be a separate pooled estimate for each disease indicator/outcome, and by type of migrant as appropriate. If there are sufficient data, there will be a separate pooled estimate of studies with adjustment and studies without adjustment. If we do not have sufficient data, adjusted and unadjusted studies will be pooled together for each disease indicator/outcome.

We will assess heterogeneity among studies by inspecting the forest plots and using the χ^2^ test for heterogeneity with a 10% level of statistical significance and using the I^2^ statistic where we interpret a value of 50% as representing moderate heterogeneity. We will assess the possibility of publication bias by evaluating funnel plot asymmetry and will also be conducted using an adjusted Egger’s regression asymmetry test as a formal statistical test for publication bias for outcomes with 10 or more studies. We will perform a leave-one-study-out sensitivity analysis to determine the stability of the results. This analysis will evaluate the influence of individual studies by estimating the pooled analyses in the absence of each study.

If there are sufficient data, we will investigate potential sources of heterogeneity, using metaregression, and incorporating the following covariates in each model: country of study, study period, type of migrant (labour, asylum seeker, refugee, undocumented, etc); setting/housing (camps, community, detention, etc); comorbidities; country of birth/origin; age and sex. To assure confidence in the results of the meta-analyses, if there are sufficient studies, we will include the following sensitivity analyses: only studies rated low risk of bias, only peer-reviewed studies, only studies that had adjustment for confounding factors and only prospective studies.

When studies cannot be combined for meta-analysis due to significant clinical heterogeneity, such as differences in participant characteristics, outcome measurements, and so on, narrative syntheses will be conducted and results of individual studies will be displayed in tables, texts and figures as appropriate, to enable a succinct summary of evidence. We will stratify the results by outcome and type of migrant and investigate heterogeneity qualitatively by exploring differences in results by country of study, study period, setting/housing, country of birth/origin, and so on, as appropriate.

#### Policy and access indicators (questions 2 and 3)

For policies (question 2), we will conduct narrative syntheses, and results of individual studies will be displayed in tables, texts and figures as appropriate, to enable a succinct summary of evidence. For access (question 3), we will use thematic analysis to group the facilitators/determinants of usage and barriers/determinants of underusage reported across studies into themes and display the results in tables, texts and figures, as appropriate. We will also stratify these results by type of migrant, country of study, study period, setting/housing and country of birth/origin, as appropriate.

All statistical analyses will be conducted in R statistical software (V.4.2.2).

### Strengths and limitations

A strength of our systematic reviews is the extensive grey literature search (including searching international organisations, ministries of health for each country, reviewing reference lists, reviewing included studies with experts, and allowing a snowballing approach to find further information). However, it may be more challenging to identify all relevant sources across all countries, and the data retrieved may not be comprehensive, of high quality, and more complicated to synthesise. To assist this process, we will document all the sources searched and data identified by source, assess the quality of the grey literature and perform sensitivity analyses for peer-reviewed versus grey literature results. Another limitation in the scope of the NCDs systematic review is that we are limiting the diseases area to diabetes only. This is to make the suite of reviews feasible; however, it is not representative of the literature on all NCDs. Once this suite of reviews is completed, we will undertake a second review on NCDs in migrant populations in the MENA region, including hypertension, cardiovascular disease, obesity and chronic kidney disease.

### Patient and public involvement

Members of the MENA Migrant Health Working Group, including clinicians and policy-makers from the Ministries of Health, IOM, WHO, Médecins du Monde and Maroc Solidarité Médico-Sociale MS2, have been involved in the design of this protocol.

### Ethics and dissemination

There are no ethical or safety issues. The seven systematic reviews will identify and summarise the relevant evidence on the data on disease burden, health outcomes, policies and barriers and facilitators to access in migrant populations in the MENA region. The findings of the systematic reviews will be summarised along with the methodological quality of the studies. Strengths and limitations of the review will be discussed and gaps in the evidence will be highlighted. The findings of these seven reviews, and those of other similar reviews or reports (if identified), will be compared.

We aim to publish each of the individual systematic reviews in peer-reviewed journals as the findings have global relevance so a peer-reviewed journal will give us this reach. In addition, we will present these findings in oral and poster presentations in relevant conferences nationally in the MENA region and internationally. We also intend to report the findings to ministries of health in Morocco, Tunisia and Egypt where, as mentioned in the introduction, we will be conducting the qualitative studies to continue the development of the MHCP-t. In addition, we will report some of these findings on our website for this project: the MENA Migrant Health project (http://www.menamigranthealth.org/). We will explore innovative ways to do public engagement work to disseminate the findings of this work to community organisations, migrant groups, NGOs and others through our growing networks in the MENA region. We envisage that they may use these findings for advocacy work and to lobby for improved service provision locally and country wide.

The data on disease burden, health outcomes, policies and facilitators and barriers to access related to migrant health in the MENA region have not been systematically reviewed, yet they have important implications for the health and well-being of migrants and the health of the local populations. These findings will be discussed with a view to better inform the understanding of data in the MENA region and the indicators we should use in the MHCP tool. The findings will also contribute more widely as a basis for future research on migrant health in the region.

## supplementary material

10.1136/bmjopen-2023-083813 online supplemental file 1
