# Supplementary material for: Defining indicators for disease burden, health outcomes, policies and barriers and facilitators to health services for migrant populations in the Middle East and North African region: a protocol for a suite of systematic reviews
Source: BMJ Open. 2024 Jul 12;14(7):e083813. doi: 10.1136/bmjopen-2023-083813 (PMC11255408; doi:10.1136/bmjopen-2023-083813)
Supplement: online supplemental file 1 [file bmjopen-14-7-s001.pdf]

Defining indicators for disease burden, health outcomes, policies, and barriers and facilitators to health services for migrant populations in the Middle East and North African region: a protocol for a suite of systematic reviews

Farah Seedat, Stella Evangelidou, Moudrick Abdellatifi, Oumnia Bouaddi, Alba Cuxart-Graell, Hassan Edries, Eman Elafef, Taha Maatoug, Anissa Ouahchi, Liv Mathilde Pampiri, Anna Deal, Sara Arias, Adel Abdelkhalek, Ahmed Hamed Arisha, Bouchra Assarag, Ibrahim Bani, Aasmaa Chaoui, Wafa Chemao-Elfihri, Kenza Hassouni, Mahmoud Hilali, , Mohamed Khalis, Wejdene Mansour, Ali Mtiraoui, Kolitha Wickramage, Dominik Zenner, Ana Requena-Mendez*, and Sally Hargreaves*, on behalf of the MENA Migrant Health Working Group

*Joint senior authors, who have contributed equally

Table of Contents

Draft search strategies ... 2

1. HIV and Hepatitis B and C ... 2
2. Malaria and NTDs ... 4
3. Tuberculosis ... 7
4. Vaccine preventable diseases ... 9
5. Maternal and neonatal health ... 12
6. Diabetes ... 15
7. Mental health ... 17

PRISMA study flow diagram ... 20

Draft search strategies for the systematic review on health indicators and policy review on policies for each disease area separately

1. HIV and Hepatitis B and C

Database(s): **Ovid MEDLINE(R) ALL** 1946 to February 01, 2023
Search Strategy:

#	Searches	Results
1	exp Human Migration/	27701
2	exp "Emigrants and Immigrants"/	15313
3	"Transients and Migrants"/	13959
4	Refugees/	12840
5	Refugee Camps/	284
6	(alien* or asile or asylum* or (border* adj2 cross*) or (countr* adj3 origin*) or diaspora or displace? or displacement* or emigrant* or emigration or expat? or expatriate? or foreigner* or foreign-born* or foreign background* or foreign population* or immigrant* or immigration or migrant* or migration or naturalized citizen* or new* arriv* or newcomer* or new-comer* or nomad* or non-citizen* or nonnative* or non-native* or nonnational or non-national or nonresident or non-resident* or resettlement* or re-settlement* or refugee* or settler* or squatter* or undocumented worker*).ti,ab,kf.	573213
7	or/1-6	586984
8	exp Middle East/	161654
9	exp Africa, Northern/	40959
10	(Abu Dhabi or Ajman or Algeri* or Arab* or Bahrain* or Bahreiin* or Dubai or Egypt* or Emirat* or Fujairah or Gaza* or Golf* or Gulf* or Ifriqiya* or Irak* or Iraq* or Jorda* or Jumhuuriiya* or Koweit* or Kuwait* or Kuwayt* or Leban* or Liban* or Liby* or Lubnan* or Maghr* or Maroc* or Maser* or Masr or Misr or MENA or Middle East* or Morocc* or North* Afric* or Oman* or Palestin* or Qatar* or Saudi* or Sharjah or Soudan* or Sudan* or Syria* or Syuri* or Tunis* or Uman* or Umm Al-Quwain or West Bank or Yemen*).ti,ab,kf.	317472
11	or/8-10	436405
12	exp Blood-Borne Infections/ or Hepatitis, Viral, Human/	428828

13	(acquired immunodeficien* syndrome* or AIDS or bloodborne disease* or blood borne disease* or bloodborne infection* or blood borne infection* or Hepatitis B or HBV or Hepatitis C or HCV or HIV or human immunodeficien* virus* or viral hepatitis or viral liver disease*).ti,ab,kf.	616455
14	or/12-13	666140
15	7 and 11 and 14	721
16	(animals not humans).sh.	5054628
17	15 not 16	719
18	limit 17 to yr="2000 -Current"	535

2. Malaria and NTDs

Database(s): **Ovid MEDLINE(R) ALL** 1946 to February 24, 2023
Search Strategy:

#	Searches	Results
1	exp Human Migration/	27722
2	exp "Emigrants and Immigrants"/	15372
3	"Transients and Migrants"/	14022
4	Refugees/	12903
5	Refugee Camps/	288
6	(alien* or asile or asylum* or (border* adj2 cross*) or (countr* adj3 origin*) or diaspora or displace? or displacement* or emigrant* or emigration or expat? or expatriate? or foreigner* or foreign-born* or foreign background* or foreign population* or immigrant* or immigration or migrant* or migration or naturalized citizen* or new* arriv* or newcomer* or new-comer* or nomad* or non-citizen* or nonnative* or non-native* or nonnational or non-national or nonresident or non-resident* or resettlement* or re-settlement* or refugee* or settler* or squatter* or undocumented worker*).ti,ab,kf.	573858
7	or/1-6	587633
8	exp Middle East/	159113
9	exp Africa, Northern/	41057
10	(Abu Dhabi or Ajman or Algeri* or Arab* or Bahrain* or Bahreiin* or Dubai or Egypt* or Emirat* or Fujairah or Gaza* or Golf* or Gulf* or Ifriqiya* or Irak* or Iraq* or Jorda* or Jumhuuriiya* or Koweit* or Kuwait* or Kuwayt* or Leban* or Liban* or Liby* or Lubnan* or Maghr* or Maroc* or Maser* or Masr or Misr or MENA or Middle East* or Morocc* or North* Afric* or Oman* or Palestin* or Qatar* or Saudi* or Sharjah or Soudan* or Sudan* or Syria* or Syuri* or Tunis* or Uman* or Umm Al-Quwain or West Bank or Yemen*).ti,ab,kf.	318043
11	or/8-10	434382
12	Neglected Diseases/	2424
13	exp Malaria/ or exp Plasmodium/	90811
14	Buruli Ulcer/ or Mycobacterium ulcerans/	1098

15	exp Dengue/ or exp Arbovirus Infections/ or exp Flavivirus Infections/	52097
16	Chikungunya Fever/	2723
17	Dracunculiasis/	907
18	exp Echinococcosis/	20591
19	exp Trematode Infections/	37974
20	Trypanosomiasis, African/ or Trypanosomiasis/ or Euglenozoa Infections/	10112
21	exp Leishmaniasis/	25341
22	exp Leprosy/ or Mycobacterium leprae/	24706
23	Elephantiasis, Filarial/ or Wuchereria bancrofti/	4066
24	Mycetoma/ or exp Dermatomycoses/	29882
25	exp Onchocerciasis/ or exp Filariasis/ or Onchocerca volvulus/	16005
26	Rabies/ or exp Rhabdoviridae Infections/	13177
27	Scabies/ or exp Ectoparasitic Infestations/	22522
28	exp Schistosomiasis/	24882
29	exp Helminthiasis/	134192
30	Snake Bites/	5405
31	exp Taeniasis/ or exp Cestode Infections/ or exp Cysticercosis/ or Neurocysticercosis/	33945
32	Trachoma/ or exp Conjunctivitis, Bacterial/	6181
33	Treponemal Infections/ or Yaws/ or Treponema pallidum/	5990
34	(neglected adj3 disease*).ti,ab,kf.	8699
35	((arbovirus or echinococcus or euglenozoa or flavivirus or nematomorpha or rhabdoviridae or schistoma or trematode or treponemal) adj3 infection*).ti,ab,kf.	3845
36	(actinomycetoma or african sleeping sickness* or african trypanosomias?s or bacterial conjunctivitis or bancroftian elephantias?s or bancroftian filarias?s or bejel* or bilharsia* or bilharzias?s or black fever or borderline tuberculoid* or breakbone fever or break bone fever or buruli ulcer or chikungunya or coenuri* or coenuros?s or coenurus cerebralis or cysticercos?s or cysticercus cellulosae or deep mycos?s or dengue* or dermatomycos?s or	350022

	dracuncul* or echinococcos?s or ectoparasitic infestation* or ectoparasitos?s or egyptian ophthalmia or eumycetoma or filarial elephantias?s or filariose or filiariasis or frambesia or guinea worm disease* or hansen* disease* or helminthias?s or hydatid cysts or hydatidos?s or hydrophobia or kalazar or kala azar or katayama fever or leishmani* or leprae or leepre or leprosies or leprosy or lymphatic filarias?s or lyssa or lyssas or madura foot or maduromycosis or malare or malaria or malayi elephantias?s or malayi filarias?s or myceetome or mycetoma or mycobacterium ulcerans or nagana or neurocysticercos?s or neuroschistosomias?s or onchocercias?s or onchocerca volvulus or onchocercose or oriental sore or plasmodium falciparum or paludisme or rabies or raby or river blindness or sarcoptic mange or scabies or schistosomias?s or schistosomal myel* or snakebite* or snake bite* or snake envenoming or taenia* or taenias?s or teeniase or trachoma* or treponema pallidum or treponematos?s or trypanosomiase or trypanosoma or trypanosomiasis or ulceere or ulcer disease* or wuchereria bancrofti or yaw or yaws or zazzabin cizon sauro).ti,ab,kf.	
37	or/12-36	543044
38	7 and 11 and 37	1064
39	(animals not humans).sh.	5062487
40	38 not 39	967
41	limit 40 to yr="2000 -Current"	692

3. Tuberculosis

Database(s): **Ovid MEDLINE(R) ALL** 1946 to February 21, 2023
Search Strategy:

#	Searches	Results
1	exp Human Migration/	27721
2	exp "Emigrants and Immigrants"/	15365
3	"Transients and Migrants"/	14005
4	Refugees/	12901
5	Refugee Camps/	288
6	(alien* or asile or asylum* or (border* adj2 cross*) or (countr* adj3 origin*) or diaspora or displace? or displacement* or emigrant* or emigration or expat? or expatriate? or foreigner* or foreign-born* or foreign background* or foreign population* or immigrant* or immigration or migrant* or migration or naturalized citizen* or new* arriv* or newcomer* or new-comer* or nomad* or non-citizen* or nonnative* or non-native* or nonnational or non-national or nonresident or non-resident* or resettlement* or re-settlement* or refugee* or settler* or squatter* or undocumented worker*).ti,ab,kf.	573334
7	or/1-6	587109
8	exp Middle East/	159051
9	exp Africa, Northern/	41052
10	(Abu Dhabi or Ajman or Algeri* or Arab* or Bahrain* or Bahreiin* or Dubai or Egypt* or Emirat* or Fujairah or Gaza* or Golf* or Gulf* or Ifriqiya* or Irak* or Iraq* or Jorda* or Jumhuuriiya* or Koweit* or Kuwait* or Kuwayt* or Leban* or Liban* or Liby* or Lubnan* or Maghr* or Maroc* or Maser* or Masr or Misr or MENA or Middle East* or Morocc* or North* Afric* or Oman* or Palestin* or Qatar* or Saudi* or Sharjah or Soudan* or Sudan* or Syria* or Syuri* or Tunis* or Uman* or Umm Al-Quwain or West Bank or Yemen*).ti,ab,kf.	317712
11	or/8-10	433998
12	exp Tuberculosis/	204795
13	(koch* disease or koch's disease or tuberculo?s*).ti,ab,kf.	248090
14	(TB adj3 (active or case* or disease* or infection* or latent)).ti,ab,kf.	18905

15	or/12-14	278936
16	7 and 11 and 15	472
17	(animals not humans).sh.	5061699
18	16 not 17	471
19	limit 18 to yr="2000 -Current"	371

4. Vaccine preventable diseases

Database(s): **Ovid MEDLINE(R) ALL** 1946 to February 13, 2023
Search Strategy:

#	Searches	Results
1	exp Human Migration/	27716
2	exp "Emigrants and Immigrants"/	15329
3	"Transients and Migrants"/	13990
4	Refugees/	12879
5	Refugee Camps/	286
6	(alien* or asile or asylum* or (border* adj2 cross*) or (countr* adj3 origin*) or diaspora or displace? or displacement* or emigrant* or emigration or expat? or expatriate? or foreigner* or foreign-born* or foreign background* or foreign population* or immigrant* or immigration or migrant* or migration or naturalized citizen* or new* arriv* or newcomer* or new-comer* or nomad* or non-citizen* or nonnative* or non-native* or nonnational or non-national or nonresident or non-resident* or resettlement* or re-settlement* or refugee* or settler* or squatter* or undocumented worker*).ti,ab,kf.	572839
7	or/1-6	586614
8	exp Middle East/	161966
9	exp Africa, Northern/	41016
10	(Abu Dhabi or Ajman or Algeri* or Arab* or Bahrain* or Bahreiin* or Dubai or Egypt* or Emirat* or Fujairah or Gaza* or Golf* or Gulf* or Ifriqiya* or Irak* or Iraq* or Jorda* or Jumhuuriiya* or Koweit* or Kuwait* or Kuwayt* or Leban* or Liban* or Liby* or Lubnan* or Maghr* or Maroc* or Maser* or Masr or Misr or MENA or Middle East* or Morocc* or North* Afric* or Oman* or Palestin* or Qatar* or Saudi* or Sharjah or Soudan* or Sudan* or Syria* or Syuri* or Tunis* or Uman* or Umm Al-Quwain or West Bank or Yemen*).ti,ab,kf.	317516
11	or/8-10	436655
12	exp Vaccines/	272039
13	exp Immunization/	205597
14	exp Immunization Programs/	15956

15	(immunostimulation* or immunisation* or immunization* or vaccin* or variolation*).ti,ab,kf.	461660
16	(immunologic* adj2 (sensiti?ation* or stimulation*)).ti,ab,kf.	603
17	Vaccine-Preventable Diseases/	225
18	Cholera/	9238
19	COVID-19/	213155
20	exp Dengue/	15622
21	Diphtheria/	6934
22	exp Hepatitis B/	64040
23	Haemophilus influenzae type b/ or Haemophilus Infections/	8717
24	exp Papillomaviridae/	37470
25	Influenza, Human/	56964
26	Measles/	14748
27	exp Meningococcal Infections/	11761
28	Mumps/	4847
29	Whooping Cough/	9095
30	exp Pneumococcal Infections/	22359
31	exp Poliomyelitis/	20598
32	Rabies/	10830
33	Rubella/	8278
34	Rotavirus Infections/	8644
35	Tetanus/	9782
36	exp Tuberculosis/	204709
37	exp Varicella Zoster Virus Infection/	19758
38	(vaccine preventable adj3 (disease* or illness* or infection*)).ti,ab,kf.	4284

39	(2019 ncov or 2019ncov or breakbone fever or break-bone fever or chickenpox* or chicken pox* or cholera* or cholerae or coqueluche or coronavirus 2 or corona virus disease 2019 or cov2 or cov 2 or covid-19 or covid19 or dengue* or diphtheri* or diphteri* or epidemic parotiti* or german measles or grippe or h1n1 or hav or haemophilus or hbv or hepa or hepatit* or herpesvirus 3 or hepb or hib or hpv or human flu or human papilloma* virus* or hydrophobia or influenza* or koch* disease or lyssa or lyssas or measles or meningit* or meningoco* or mumps or ncov or neisseria meningitidis or neonatal calf diarrhea virus* or new corona virus* or new coronavirus* or novel corona virus* or novel coronavirus* or oreillons or pachymeningiti* or papillomavir* or pertuss* or pfeiffer* bacillus or pneumococcal or pneumoniae or polio* or rabies or rotavirus* or rougeol* or rubella* or rubeol* or sars-cov-2 or sars2 or tdap or tdp or tetani or tetanus* or three day measle* or tuberculos* or varicella or whooping cough*).ti,ab,kf.	1340987
40	or/12-39	1744210
41	7 and 11 and 40	1725
42	(animals not humans).sh.	5059283
43	41 not 42	1682
44	limit 43 to yr="2000 -Current"	1397

5. Maternal and neonatal health

Database(s): **Ovid MEDLINE(R) ALL** 1946 to March 23, 2023
Search Strategy:

#	Searches	Results
1	exp Human Migration/	27742
2	exp "Emigrants and Immigrants"/	15413
3	"Transients and Migrants"/	14073
4	Refugees/	12961
5	Refugee Camps/	287
6	(alien* or asile or asylum* or (border* adj2 cross*) or (countr* adj3 origin*) or diaspora or displace? or displacement* or emigrant* or emigration or expat? or expatriate? or foreigner* or foreign-born* or foreign background* or foreign population* or immigrant* or immigration or migrant* or migration or naturalized citizen* or new* arriv* or newcomer* or new-comer* or nomad* or non-citizen* or nonnative* or non-native* or nonnational or non-national or nonresident or non-resident* or resettlement* or re-settlement* or refugee* or settler* or squatter* or undocumented worker*).ti,ab,kf.	576748
7	or/1-6	590527
8	exp Middle East/	159720
9	exp Africa, Northern/	41145
10	(Abu Dhabi or Ajman or Algeri* or Arab* or Bahrain* or Bahreiin* or Dubai or Egypt* or Emirat* or Fujairah or Gaza* or Golf* or Gulf* or Ifriqiya* or Irak* or Iraq* or Jorda* or Jumhuuriiya* or Koweit* or Kuwait* or Kuwayt* or Leban* or Liban* or Liby* or Lubnan* or Maghr* or Maroc* or Maser* or Masr or Misr or MENA or Middle East* or Morocc* or North* Afric* or Oman* or Palestin* or Qatar* or Saudi* or Sharjah or Soudan* or Sudan* or Syria* or Syuri* or Tunis* or Uman* or Umm Al-Quwain or West Bank or Yemen*).ti,ab,kf.	319881
11	or/8-10	436540
12	Maternal Health/ or Maternal Welfare/	8804
13	exp Maternal Health Services/ or Maternal-Child Health Centers/	58953
14	Maternal Mortality/ or Maternal Death/	11807

15	Hypertension, Pregnancy-Induced/ or Pre-Eclampsia/	38475
16	Diabetes, Gestational/	14923
17	Depression, Postpartum/	7293
18	Postpartum Hemorrhage/	8327
19	Anemia, Iron-Deficiency/	11555
20	Pregnancy Complications, Infectious/ or Puerperal Infection/	44906
21	Pregnancy/	978234
22	Gravidity/	1308
23	Pregnancy, High-Risk/ or Pregnancy, Unplanned/ or Pregnancy, Unwanted/	9870
24	Pregnant Women/	13892
25	Pregnancy Trimester, Third/	15768
26	Peripartum Period/	1713
27	exp Delivery, Obstetric/ or Labor, Obstetric/ or Labor Onset/ or exp Labor Presentation/ or "Trial of Labor"/ or Uterine Contraction/	121577
28	Birth Setting/ or Home Childbirth/ or Natural Childbirth/ or Term Birth/	8431
29	Pregnancy Outcome/ or Abortion, Spontaneous/ or Stillbirth/ or Abortion, Incomplete/ or Embryo Loss/ or Abortion, Threatened/	84632
30	Uterine Cervical Incompetence/	1571
31	Obstetric Labor Complications/ or Dystocia/ or Premature Birth/	41246
32	Infant Health/	1251
33	exp Infant Mortality/	31664
34	exp Infant, Low Birth Weight/	38613
35	exp Infant, Premature/	64190
36	(birth* or child bearing or childbearing or childbirth* or expect* mother* or gestation* or gravidit* or multigravidit* or nulligravidit* or obstetric deliver* or pregnan* or primigravidit*).ti,ab,kf.	968064

37	((antenatal* or ante natal* or maternal or perinatal* or peri natal* or peripartum or peri partum or postnatal or post natal or postpartum or post partum or puerperium) adj3 (care or clinic* or healthcare or service*)).ti,ab,kf.	42845
38	(abortion* or breech presentation* or dystocia* or embryo death or embryo loss or embryo resorption or fetal presentation* or incompetent cervi* or labor complication* or labor onset* or labor presentation* or labor trial or obstetric labor or prematurity or "trial of labor" or uterine contraction* or uterine cervi* incompetence).ti,ab,kf.	111733
39	((prematur* or preterm or pre-term) adj3 (baby or babies or child* or deliver* or infant* or neonat* or newborn*)).ti,ab,kf.	95191
40	((low or small) adj3 (birthweight* or birth weight* or gestational age)).ti,ab,kf.	51845
41	(an?emia* or fetal macrosomia* or gestational diabetes or gestational hypertension or postpartum sepsis or pre-eclampsia or preeclampsia or puerperal infection* or stillbirth*).ti,ab,kf.	240331
42	((postnatal or post natal or postpartum or post partum) adj2 (depress* or dysphoria* or h?emorrhage*)).ti,ab,kf.	18564
43	((baby or babies or infant* or maternal* or neonat* or newborn* or peri natal* or perinatal*) adj3 (death* or health* or mortalit*)).ti,ab,kf.	135076
44	or/12-43	1658543
45	7 and 11 and 44	1881
46	(animals not humans).sh.	5071308
47	45 not 46	1843
48	limit 47 to yr="2000 -Current"	1308

6. Diabetes

Database(s): **Ovid MEDLINE(R) ALL** 1946 to May 25, 2023
Search Strategy:

#	Searches	Results
1	exp Human Migration/	27778
2	exp "Emigrants and Immigrants"/	15514
3	"Transients and Migrants"/	14172
4	Refugees/	13100
5	Refugee Camps/	289
6	(alien* or asile or asylum* or (border* adj2 cross*) or (countr* adj3 origin*) or diaspora or displace? or displacement* or emigrant* or emigration or expat? or expatriate? or foreigner* or foreign-born* or foreign background* or foreign population* or immigrant* or immigration or migrant* or migration or naturalized citizen* or new* arriv* or newcomer* or new-comer* or nomad* or non-citizen* or nonnative* or non-native* or nonnational or non-national or nonresident or non-resident* or resettlement* or re-settlement* or refugee* or settler* or squatter* or undocumented worker*).ti,ab,kf.	583255
7	or/1-6	597041
8	exp Middle East/	160899
9	exp Africa, Northern/	41353
10	(Abu Dhabi or Ajman or Algeri* or Arab* or Bahrain* or Bahreiin* or Dubai or Egypt* or Emirat* or Fujairah or Gaza* or Golf* or Gulf* or Ifriqiya* or Irak* or Iraq* or Jorda* or Jumhuuriiya* or Koweit* or Kuwait* or Kuwayt* or Leban* or Liban* or Liby* or Lubnan* or Maghr* or Maroc* or Maser* or Masr or Misr or MENA or Middle East* or Morocc* or North* Afric* or Oman* or Palestin* or Qatar* or Saudi* or Sharjah or Soudan* or Sudan* or Syria* or Syuri* or Tunis* or Uman* or Umm Al-Quwain or West Bank or Yemen*).ti,ab,kf.	323995
11	or/8-10	441338

12	exp Diabetes Mellitus/	504402
13	exp Insulin Resistance/	98730
14	(diabet* or insulin* or metabolic syndrome* or cardiometabolic syndrome* or dysmetabolic syndrome* or reaven syndrome*).ti,ab,kf.	1034882
15	or/12-14	1092851
16	7 and 11 and 15	599
17	(animals not humans).sh.	5090355
18	16 not 17	590
19	limit 18 to yr="2000 -Current"	541

7. Mental health

Database(s): **Ovid MEDLINE(R) ALL** 1946 to June 08, 2023
Search Strategy:

#	Searches	Results
1	exp Human Migration/	27795
2	exp "Emigrants and Immigrants"/	15560
3	"Transients and Migrants"/	14191
4	Refugees/	13137
5	Refugee Camps/	291
6	(alien* or asile or asylum* or (border* adj2 cross*) or (countr* adj3 origin*) or diaspora or displace? or displacement* or emigrant* or emigration or expat? or expatriate? or foreigner* or foreign-born* or foreign background* or foreign population* or immigrant* or immigration or migrant* or migration or naturalized citizen* or new* arriv* or newcomer* or new-comer* or nomad* or non-citizen* or nonnative* or non-native* or nonnational or non-national or nonresident or non-resident* or resettlement* or re-settlement* or refugee* or settler* or squatter* or undocumented worker*).ti,ab,kf.	584365
7	or/1-6	598153
8	exp Middle East/	161066
9	exp Africa, Northern/	41399
10	(Abu Dhabi or Ajman or Algeri* or Arab* or Bahrain* or Bahreiin* or Dubai or Egypt* or Emirat* or Fujairah or Gaza* or Golf* or Gulf* or Ifriqiya* or Irak* or Iraq* or Jorda* or Jumhuuriiya* or Koweit* or Kuwait* or Kuwayt* or Leban* or Liban* or Liby* or Lubnan* or Maghr* or Maroc* or Maser* or Masr or Misr or MENA or Middle East* or Morocc* or North* Afric* or Oman* or Palestin* or Qatar* or Saudi* or Sharjah or Soudan* or Sudan* or Syria* or Syuri* or Tunis* or Uman* or Umm Al-Quwain or West Bank or Yemen*).ti,ab,kf.	324777
11	or/8-10	442219

12	Mental Health/ or exp Mental Disorders/ or Depression/ or Anxiety/ or Resilience, Psychological/	1608207
13	(mental adj2 (disorder* or distress or health or ill-being or illbeing or illness* or ill health or illhealth or instabilit* or symptom* or wellbeing or well-being or wellness)).ti,ab,kf.	299115
14	((psychiatric or psychologic*) adj3 (disorder* or distress or ill-being or illbeing or ill health or illhealth or illness* or problem* or resilienc* or symptom*)).ti,ab,kf.	141346
15	((drug* or heroin or inhalant* or marijuana or morphine or opium or substance*) adj3 (abuse* or addict* or dependenc* or habituation*)).ti,ab,kf.	91981
16	(alcoholic intoxication* or alcoholic psychos* or alcoholism or alcohol withdrawal delirium or binge drinking or opiate overdose or substance-induced psychos*).ti,ab,kf.	39664
17	((alcoholic korsakoff or asperger* or battered child or capgras or creutzfeldt-jakob or female athlete triad or jet lag or kleine-levin or kluver-bucy or munchausen or neonatal abstinence or night eating or restless legs or rumination or substance withdrawal or tourette*) adj3 syndrome*).ti,ab,kf.	16164
18	((adjustment or affective or alcohol-related or amphetamine-related or attention deficit or auditory perceptual or autistic or autism or bipolar or body dysmorphic or child behavio?r* or (pervasive adj2 development*) or cocaine-related or cognition or combat or communication or conduct or consciousness or excessive somnolence or disruptive behavio?r* or dissociative or "drug use" or eating or elimination or factitious or feeding or gender or hoarding or hyperactivity or impulse control or mood or motor or motor skill* or narcotic-related or neurocognitive or neurodevelopmental or neurotic or obsessive-compulsive or opioid-related or panic or paranoid or paraphilic or personality or phobic or psychotic or sexual or sleep* or sleep wake or somatoform or stress* or substance-related or tic or "tobacco use" or trauma) adj3 disorder*).ti,ab,kf.	346123
19	(agoraphobia or alzheimer disease or amnesia or anorexia nervosa or anxiety or bulimia nervosa or cataplexy or chemotherapy-related cognitive impairment or cognitive dysfunction* or delirium or delusional parasitosis or dementia* or depression* or developmental disabilit* or diabulimia or "diffuse neurofibrillary tangles with calcification" or diurnal enuresis or dyscalculia or dyslexia or dyspareunia or dyssomnia* or emergence delirium or encopresis or enuresis or erectile dysfunction or exhibitionism or firesetting behavior* or food addict* or frontotemporal lobar degeneration or	1001530

	gambling or gender dysphoria* or globus sensation or historical trauma* or huntington disease or hypochondriasis or hysteria* or idiopathic hypersomnia or intellectual disabilit* or kinesiophobia* or learning disabilit* or lewy body disease or masochism or morgellons disease or mutism or narcolepsy or neurasthenia or neurocirculatory asthenia or neurosis or neuroses or night terror* or nocturnal enuresis or nocturnal paroxysmal dystonia or orthorexia nervosa or parasomnia* or pedophilia or phencyclidine abuse* or pica or "pick disease of the brain" or postoperative cognitive complication* or premature ejaculation or primary progressive aphasia or primary progressive nonfluent aphasia* or psychiatric fetishism or psychological sexual dysfunction* or psychological trauma* or pure alexia or "relative energy deficiency in sport" or rem sleep parasomnia* or sadism or schizophrenia or sexual trauma* or sleep bruxism or sleep deprivation* or sleep paralysis or sluggish cognitive tempo or social phobia* or somnambulism or transvestism or trichotillomania or vaginismus or voyeurism or wernicke encephalopathy).ti,ab,kf.	
20	or/12-19	2205440
21	7 and 11 and 20	2636
22	(animals not humans).sh.	5094204
23	21 not 22	2629
24	limit 23 to yr="2000 -Current"	2326

PRISMA study flow diagram

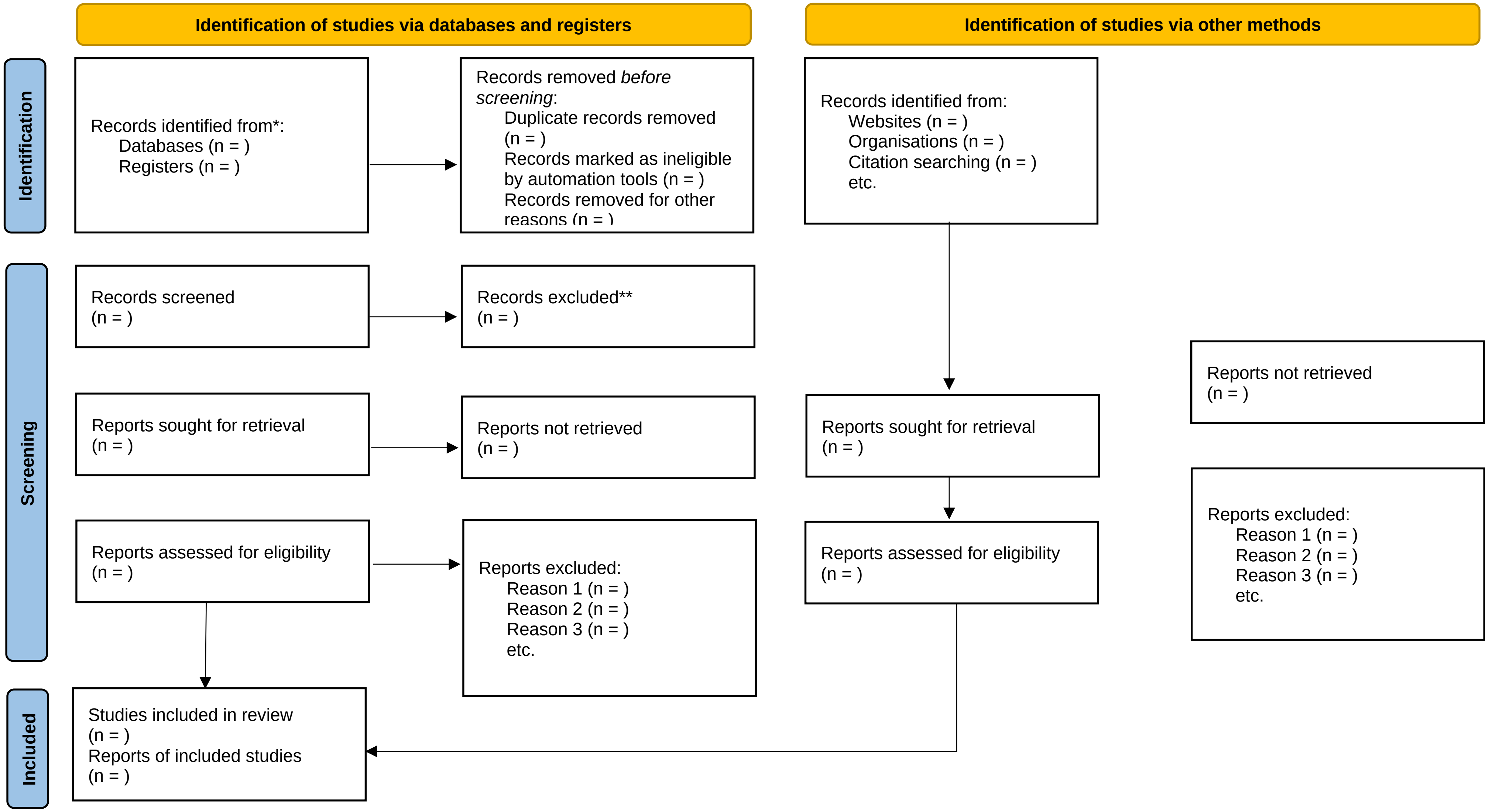

From: Page MJ, McKenzie JE, Bossuyt PM, Boutron I, Hoffmann TC, Mulrow CD, et al. The PRISMA 2020 statement: an updated guideline for reporting systematic reviews. BMJ 2021;372:n71. doi: 10.1136/bmj.n71. For more information, visit: http://www.prisma-statement.org/
