# Supplementary material for: Defining indicators for disease burden, health outcomes, policies and barriers and facilitators to health services for migrant populations in the Middle East and North African region: a protocol for a suite of systematic reviews
Source: BMJ Open. 2024 Jul 12;14(7):e083813. doi: 10.1136/bmjopen-2023-083813 (PMC11255408; doi:10.1136/bmjopen-2023-083813)
Supplement: Supplementary file 2 [file bmjopen-2023-083813.reviewer_comments.pdf]

PEER REVIEW HISTORY

BMJ Open publishes all reviews undertaken for accepted manuscripts. Reviewers are asked to complete a checklist review form (**http://bmjopen.bmj.com/site/about/resources/checklist.pdf**) and are provided with free text boxes to elaborate on their assessment. These free text comments are reproduced below.

ARTICLE DETAILS

**TITLE (PROVISIONAL)**	Defining indicators for disease burden, health outcomes, policies, and barriers and facilitators to health services for migrant populations in the Middle East and North African region: a protocol for a suite of systematic reviews
**AUTHORS**	Seedat, Farah; Evangelidou, Stella; Abdellatifi, Moudrick; Bouaddi, Oumnia; Cuxart-Graell, Alba; Edries, Hassan; Elafef, Eman; Maatoug, Taha; Ouahchi, Anissa; Mathilde Pampiri, Liv; Deal, Anna; Arias, Sara; Abdelkhalek, Adel; Arisha, Ahmed; Assarag, Bouchra; BANI, IBRAHIM AHMED; Chaoui, Aasmaa; Chemao-Elfihri, Wafa; HASSOUNI, Kenza; Hilali, Mahmoud; Khalis, Mohamed; Mansour, Wejdene; Mtiraoui, Ali; Wickramage, KolithaEditorial Board Member; Zenner, D; Requena-Mendez, Ana; Hargreaves, Sally; Migrant Health Working Group, MENA

VERSION 1 – REVIEW

**REVIEWER**	Al-Maadheed, Mohammed<br>University College London, Centre of Metabolism & Inflammation, Division of Medincine
**REVIEW RETURNED**	31-Aug-2023

**GENERAL COMMENTS**	This is an interesting manuscript setting out a protocol to define, in migrants' populations of the MENA region, indicators for the burden of disease, measure of outcomes, policies and barriers to access healthcare services, using a collection of systemic reviews and other 'grey' material. Such a study is warranted as there is paucity of data related to the health of, and healthcare availability to, migrants living in this area. The research question and study objective are clearly defined, with a well written abstract.<br>However, there are concerns related to the appropriateness of the study design to answer the research question. Specifically, there are three major drawbacks to this study:<br>A. The study population: The migrants living in the countries of the MENA region should not be grouped as one. The MENA countries are three rather disparate groups with very different types of migrants, who have different healthcare needs, and the available facilities in the host countries also varied. The first group include the majority of the GCC countries, specifically Kuwait, Saudi Arabia, United Arab Emirates, Oman, Bahrain, and Qatar, these are very affluent counties mainly dealing with an influx of temporary, economic migrants from the neighboring countries, the majority from the Indian sub-continent. The second group are the war-torn countries, namely Libya, Occupied Palestinian Territories, Sudan, Syria and Yemen, facing significant internal displacement, refugees, with destruction of healthcare infrastructure. Lastly, the countries that are a mixture of the above two, which include Morocco, Egypt, Algeria, Iraq, Jordan and Tunisia, where there is

	still significant internal displacement, along with refugees and migrants from neighboring countries.<br>The make-up of the migrants in each of these groups are different, with significant differences in their healthcare needs, the host countries also vary significantly in their ability to meet these needs of the migrants adequately.<br>B. Choice of diseases: the ones described/selected are rather limited, and also significantly dependent on the three groups of migrants. For example, non-communicable diseases (NCDs), specifically hypertension, CVD, metabolic dysfunction (dyslipidemia), obesity and chronic kidney disease, as well as sexually transmitted diseases, are the main reasons for morbidity and mortality amongst the migrants in the GCC countries. Only diabetes is included in this study. However, work related injuries and road traffic accidents, along with the NCDs, account for most of their healthcare needs. Infectious diseases are of much less concern in the migrants in these countries. These are likely to be of major concern in the war-torn countries.<br>C. Sources of information and access to this information: While publications from these countries can certainly help provide some of this information, much must be obtained from the other sources, such as Red Crescent Societies, which serve many of these countries. Only Lebanon has a Red Cross Society. ICRC mainly operates in the war zones.<br>With the concerns outlined above the methods described in this protocol may not be sufficient to allow the study to be repeated. Also, the outcomes are not clearly defined. Again, this relates to the disparity in the migrants in this region and need to be readdressed. Because of the issues raised above the discussion and conclusions need to be revisited as do the limitations of the study.<br>Research ethics and statistics are described adequately. The references are up-to-date and appropriate and presented clearly. There are no concerns related to plagiarism, conflicts of interest and the manuscript is written and of a standard acceptable for publication.

**REVIEWER**	Kjøllesdal, Marte<br>Norwegian Institute of Public Health
**REVIEW RETURNED**	17-Oct-2023

**GENERAL COMMENTS**	Defining indicators for disease burden, clinical outcomes, policies, and barriers to health services for migrant populations in the Middle East and North African region: a protocol for a suite of systematic reviews<br><br>Thanks for the possibility to read this protocol for a an interesting, extensive and important review work. The protocol describes the search strategy for seven systematic reviews that aim to identify, appraise, and synthesise the available evidence on disease burden among migrants and relevant policies in the MENA region.<br><br>The introduction is in general well-written and argues well for the need of systematic reviews. The choice of databases and sources of grey information seems seasonable, and also the choice of guidelines to follow. The choice of search terms, however, are not well argued, and seems to include overly detailed (and wrong) search words some places and miss out on important diagnoses in

	other places. Moreover, the research questions with belonging indicators are not clear.<br><br>More detailed comments are provided below:<br><br>Introduction:<br><br>“The current conflict in Sudan has intensified the situation, with reports of over 1.4 million people becoming newly displaced (approximately 476,811 fleeing to neighbouring countries)” 476,811 are not approximately, but rather very (too) exact.<br><br>The definition of a migrant: would it be better to phrase your own definition, as e.g. the example here being international student might not be especially relevant to you?<br><br>Methods and Research questions:<br><br>What data are available on the disease indicators related to each of the seven disease areas in migrant<br>populations in the MENA region?<br>Objectives:<br>a. Synthesise the burden of TB, HIV, hepatitis B and C, malaria, neglected tropical diseases,<br>diabetes, mental health, maternal and neonatal health conditions, and VPDs in migrant<br>populations in the MENA region.<br>b. Synthesise the (intermediate and final) clinical outcomes of TB, HIV, hepatitis B and C, malaria,<br>neglected tropical diseases, diabetes, mental health, maternal and neonatal health conditions, and<br>VPDs in migrant populations in the MENA region.<br>c. Examine the quality of evidence on the burden, clinical process, and final health outcomes of TB,<br>HIV, hepatitis B and C, malaria, neglected tropical diseases, diabetes, mental health, maternal and<br>neonatal health conditions, and VPDs in migrant populations in the MENA region.<br><br>It is not clear what is the difference between objective a and b. In the method it reads “For disease indicators (question 1), we will include papers that are on the burden (e.g., prevalence or incidence) or intermediate (e.g., coverage or completion of interventions such as screening or treatment) or final (e.g. morbidity, mortality, quality of life) clinical outcomes for TB, HIV, hepatitis B and C, malaria, neglected tropical diseases, diabetes, mental health, maternal and neonatal health conditions, and VPDs in migrant populations in the MENA region.” What would be the difference between prevalence of a disease and morbidity (e.g. between prevalence of depression and morbidity related to depression)? And would not intervention coverage be an indicator of service use rather than an indicator of disease? These terms and indicators need to be clarified.<br><br>Would not c be an integrated part of objective a and b?<br><br>Research question 2, form the methods:<br>“For the policy-related data (question 2), we will include papers that contain a description of the policies themselves, uptake of the

	health services mentioned in the policies and determinants of any under-usage, and facilitators or barriers in accessing the health services mentioned in the policies for the diseases in migrant populations in the MENA region. Definitions for migrant and the MENA region are described in panel 1." What about use of regular health services and barriers to that? Most health services will probably be regular services not described in policies regarding migrants especially?<br><br>Search strategies: "An iterative procedure was used, with input from all authors including an information scientist, recommended search filters, and previous reviews." Can you explain what input from recommended search filters could be?<br><br>It can be considered to start the search even later than 2000, as almost 25 years will have passed when the results are ready, and earlier information may not be especially relevant anymore.<br><br>You have no language restriction: What is the plan is you come across a study in a language none of the authors have competencies in?<br><br>Why have you chosen diabetes and not other NCDs? You have included very many different VPDs, so why not include other NCDs like hypertension, CVDs, stroke or cancer? This would have been just as relevant, thus this choice needs an explanation. And what about including hyperglycemia as a search term with diabetes?<br><br>For mental health: You have included some more general search terms like "mental health" and mental disorders" and some common mental disorders like "anxiety" and depression". You have also a lot of diagnoses which is not common/correct to include as a mental disorder, like "diffuse neurofibrillary tangles with calcification", "relative energy deficiency in sport", "vaginisimus" and erectile dysfunction", and others which seems of little relevance, like "kinesiophobia*. Other terms which I would consider much more relevant, such as "post traumatic stress syndrome" is not included. Moreover, various substance use disorders are included as mental health (which it is not, although they might co-appear) and also tobacco use. I would suggest to revise included search terms and eliminate all those not relevant, and also to include an own search for substance use disorders.<br><br>In general; the choice of search terms seems a bit random, and would benefit from better clarification and arguments.<br><br>Table 2: same comment as above, it is not clear what is really the difference between "burden" and "final clinical outcome" and "intermediate clinical outcome". This needs to be explained (and maybe re-named?)<br><br>Should the flow chart been modified to better reflect the process of including grey literature and the extensive search in policy documents?<br><br>Ethics and dissemination:

	“We also intend to report the findings to ministries of health in Morocco, Tunisia, and Sudan<br>where we will be conducting the qualitative studies to continue the development of the MHCP-t.” The qualitative studies referred to have not been described in the article?

VERSION 1 – AUTHOR RESPONSE

No	Comment	Response
**Reviewer 1**		
1	This is an interesting manuscript setting out a protocol to define, in migrants' populations of the MENA region, indicators for the burden of disease, measure of outcomes, policies and barriers to access healthcare services, using a collection of systemic reviews and other 'grey' material. Such a study is warranted as there is paucity of data related to the health of, and healthcare availability to, migrants living in this area. The research question and study objective are clearly defined, with a well written abstract.[1]	We thank you for your comments.
2	However, there are concerns related to the appropriateness of the study design to answer the research question. Specifically, there are three major drawbacks to this study:<br>A. The study population: The migrants living in the countries of the MENA region should not be grouped as one. The MENA countries are three rather disparate groups with very different types of migrants, who have different healthcare needs, and the available facilities in the host countries also varied. The first group include the majority of the GCC countries, specifically Kuwait, Saudi Arabia, United Arab Emirates, Oman, Bahrain, and Qatar, these are very affluent counties mainly dealing with an influx of temporary, economic migrants from the neighboring countries, the majority from the Indian sub-continent. The second group are the war-torn countries, namely Libya, Occupied Palestinian Territories, Sudan, Syria and Yemen, facing significant internal displacement, refugees, with destruction of healthcare infrastructure. Lastly, the countries that are a mixture of the above two, which include Morocco, Egypt, Algeria, Iraq, Jordan and Tunisia, where	We agree with the reviewer that migrants are a heterogenous and diverse group, as mentioned in our introduction. We also agree that different migrant groups may have different needs and healthcare access. We think it is important to explore the burden of diseases and healthcare access across all these migrant groups to investigate what similarities and differences exist between the groups. Therefore, we are including articles on all types of migrants in line with the International Organisation of Migration's (IOM's) definition, and we will then explore the similarities and differences between the migrant groups in our data synthesis. We will explore difference across the three types of countries in the MENA region mentioned by the reviewer. As mentioned in our manuscript, we will stratify the results by migrant type, where possible. We have added the following additional sentences about this in the methods on pages 17-18:<br><br>*"There will be a separate pooled estimate for each disease indicator / outcome, and by type of migrant as appropriate."* (Disease indicators, question 1, page 17)

[1] Blah et al.

No	Comment	Response
	there is still significant internal displacement, along with refugees and migrants from neighboring countries.<br>The make-up of the migrants in each of these groups are different, with significant differences in their healthcare needs, the host countries also vary significantly in their ability to meet these needs of the migrants adequately.[1]	*"We will stratify the results by outcome and type of migrant and investigate heterogeneity qualitatively by exploring differences in results by country of study, study period, setting/housing, country of birth/origin, etc., as appropriate."* (when studies cannot be combined for meta-analysis due to significant clinical heterogeneity, page 18)<br><br>*We will also stratify these results by outcome and type of migrant as appropriate.* (policies and access, questions 2 and 3, page 18)
3	B. Choice of diseases: the ones described/selected are rather limited, and also significantly dependent on the three groups of migrants. For example, non-communicable diseases (NCDs), specifically hypertension, CVD, metabolic dysfunction (dyslipidemia), obesity and chronic kidney disease, as well as sexually transmitted diseases, are the main reasons for morbidity and mortality amongst the migrants in the GCC countries. Only diabetes is included in this study. However, work related injuries and road traffic accidents, along with the NCDs, account for most of their healthcare needs. Infectious diseases are of much less concern in the migrants in these countries. These are likely to be of major concern in the war-torn countries.	In line with the rationale above, we wanted to cast a wide net across disease areas that might be important to the different migrant groups, therefore, we have tried to cover as many key communicable and non-communicable diseases as possible. We acknowledge that for non-communicable diseases we have only included maternal and neonatal health conditions, mental health, and diabetes. We initially planned to look at multiple non-communicable diseases, however, the number of hits was unmanageable, so we chose all maternal and neonatal health conditions, all mental health conditions, and diabetes. We chose diabetes as, in our scoping for this review, we found diabetes to be the disease of most concern for migrants in the region (which we reference in the introduction).<br><br>We do agree it is important to look at other conditions so we are pleased to inform you that while we will include diabetes at this first stage, in the next stage we will do a systematic review on other NCDs, including hypertension, CVD, obesity and CKD. We have added the following sentence about this choice and the next steps in the methods (page 11) and added a limitation section at the end of the methods (pages 18-19), acknowledging this, in addition to other limitations:

No	Comment	Response
		".. for NCDs, we have chosen diabetes for this first stage. In the next stage we will do a wider systematic review on NCDs in migrant populations in the MENA region, including hypertension, CVD, obesity, and CKD." (page 11)<br><br>*"Another limitation in the scope of the NCDs systematic review is that we are limiting the diseases area to diabetes only. This is to make the suite of reviews feasible, however, it is not representative of the literature on all NCDs. Once this suite of reviews is completed, we will undertake a second review on NCDs in migrant populations in the MENA region, including hypertension, CVD, obesity, and CKD."* (pages 18-19)
4	C. Sources of information and access to this information: While publications from these countries can certainly help provide some of this information, much must be obtained from the other sources, such as Red Crescent Societies, which serve many of these countries. Only Lebanon has a Red Cross Society. ICRC mainly operates in the war zones.	We agree that much information may have to be obtained from grey literature sources, but we can only confirm this based on evidence once we have reviewed the literature, which is one of the purposes of this review.<br><br>We think that the extensive grey literature search (including searching international organisations, ministries of health for each country, reviewing reference lists, reviewing included studies with experts, and allowing a snowballing approach to find further information) is a strength of the project.<br><br>Once we have the results, we will be able to confirm how much evidence has been peer-reviewed and how much is published in other sources.
5	With the concerns outlined above the methods described in this protocol may not be sufficient to allow the study to be repeated.	We understand your concern, but we humbly disagree. The study can be repeated using the electronic searches and categorising the available data by migration and outcome. The grey literature search from any systematic review is always difficult to repeat as it requires searching of multiple sources in different ways. However, in our manuscript we have stated the organisations we will be searching initially, and this can be repeated. Furthermore, upon completion of these systematic reviews we will report every single organisation searched through the snowballing approach of organisation so this will also be repeatable. We have added a sentence in the methods to describe this:

No	Comment	Response
		"We will report every organisation we search in the write up of the review." (page 10)
6	Also, the outcomes are not clearly defined. Again, this relates to the disparity in the migrants in this region and need to be readdressed.	We thank you for flagging this lack of clarity in the definitions. We have discussed the outcomes and have re-categorised as follows (see pages 6-7, 10,12-15):<br><br>a. Indicators:<br>- Burden outcomes (e.g., prevalence or incidence)<br>- Invention outcomes (e.g., uptake and coverage of screening or treatment, treatment success)<br>- Intermediate outcomes (e.g., severity of disease, prognosis)<br>- Final outcomes (e.g., mortality, quality of life)<br>b. Policies<br>c. Barriers and facilitators
7	Because of the issues raised above the discussion and conclusions need to be revisited as do the limitations of the study.	We have added a sub-section acknowledging the limitations of the review at the end of the methods section on pages 18-19:<br><br>***"Strengths and Limitations***<br><br>*A strength of our systematic reviews is the extensive grey literature search (including searching international organisations, ministries of health for each country, reviewing reference lists, reviewing included studies with experts, and allowing a snowballing approach to find further information). However, these data may be more challenging to identify all relevant sources across all countries and the data retrieved may not be comprehensive, of high quality and more complicated to synthesise. To assist this process, we will document all the sources searched and data identified by source, assess the quality of the grey literature, and perform sensitivity analyses for peer-reviewed versus grey literature results. Another limitation in the scope of the NCDs systematic review is that we are limiting the diseases area to diabetes only. This is to make the suite of reviews feasible, however, it is not representative of the literature on all NCDs. Once this suite of reviews is completed, we will undertake a second review on*

No	Comment	Response
		NCDs in migrant populations in the MENA region, including hypertension, CVD, obesity, and CKD."
8	Research ethics and statistics are described adequately. The references are up-to-date and appropriate and presented clearly. There are no concerns related to plagiarism, conflicts of interest and the manuscript is written and of a standard acceptable for publication.	We thank you for your comments.
**Reviewer 2**		
9	Thanks for the possibility to read this protocol for a an interesting, extensive and important review work. The protocol describes the search strategy for seven systematic reviews that aim to identify, appraise, and synthesise the available evidence on disease burden among migrants and relevant policies in the MENA region.	We thank you for your comments.
10	The introduction is in general well-written and argues well for the need of systematic reviews. The choice of databases and sources of grey information seems seasonable, and also the choice of guidelines to follow.	We thank you for your comments.
11	The choice of search terms, however, are not well argued, and seems to include overly detailed (and wrong) search words some places and miss out on important diagnoses in other places.	Much thought and input went into the construction of the search strategies. We had an expert information scientist who constructed the search strategies combined with topic experts who reviewed the search strategies until we reached an agreement.
12	Moreover, the research questions with belonging indicators are not clear.	We thank you for flagging this point. We have discussed this and are happy to re-define the research questions and indicators as follows (pages 6-7). The re-categorisation of indicators is also shown above in our response to question 6.<br><br>*"*<br><br>1. *What data are available on the disease indicators related to each disease area in migrant populations in the MENA region?*<br>*Objectives:*

No	Comment	Response
		a. *Synthesise and appraise data on the burden (e.g. prevalence or incidence) of TB, HIV, hepatitis B and C, malaria, NTDs, diabetes, mental health, maternal and neonatal health conditions, and VPDs in migrant populations in the MENA region.*<br>b. *Synthesise and appraise data on intervention outcomes (e.g., uptake and coverage of vaccination, screening or treatment, treatment success) related to TB, HIV, hepatitis B and C, malaria, NTDs, diabetes, mental health, maternal and neonatal health, and VPDs in migrant populations in the MENA region.*<br>c. *Synthesise and appraise data on the intermediate (e.g., symptoms, severity, or prognosis / long-term morbidity of disease) and final (e.g., mortality, quality of life) health outcomes of TB, HIV, hepatitis B and C, malaria, NTDs, diabetes, mental health, maternal and neonatal health conditions, and VPDs in migrant populations in the MENA region.*<br><br>2. *What is the policy response for each disease area related to migrant populations in the MENA region?*<br>*Objective:*<br><br>a. *Synthesise and appraise the prevention and/or treatment policies for TB, HIV, hepatitis B and C, malaria, NTDs, diabetes, mental health, maternal and neonatal health, and VPDs in migrant populations in the MENA region.*<br><br>3. *What are the barriers and facilitators in accessing health services for each disease area for migrant populations in the MENA region?*<br>*Objective:*<br><br>a. *Synthesise and appraise the evidence on the barriers and facilitators for accessing prevention and/or treatment services for TB, HIV, hepatitis B and C, malaria, NTDs, diabetes, mental health, maternal and neonatal health, and VPDs in migrant populations in the MENA region."*
13	Introduction:<br>"The current conflict in Sudan has intensified the situation, with reports of over 1.4 million people becoming newly displaced (approximately 476,811 fleeing to neighbouring countries)" 476,811 are not approximately, but rather very (too) exact.	We have updated this figure to December 2023 and added in the number from Gaza:<br><br>*"over 1.38 million fleeing to neighbouring countries"* (page 3).

No	Comment	Response
14	The definition of a migrant: would it be better to phrase your own definition, as e.g., the example here being international student might not be especially relevant to you?	We spent a long time discussing the definition of migrant with all the members of the consortium across many countries in the Middle East and North Africa and experts on migration research. It was agreed that the IOM definition is the most appropriate as there is currently no agreed definition of migrant and the IOM definition has the most credibility. As mentioned above in question 2, we intentionally kept a broad definition as it is important to explore the literature on all different types of migrants, including international students, to see what the differences are between the groups.
15	Methods and Research questions:<br><br>It is not clear what is the difference between objective a and b. In the method it reads “For disease indicators (question 1), we will include papers that are on the burden (e.g., prevalence or incidence) or intermediate (e.g., coverage or completion of interventions such as screening or treatment) or final (e.g. morbidity, mortality, quality of life) clinical outcomes for TB, HIV, hepatitis B and C, malaria, neglected tropical diseases, diabetes, mental health, maternal and neonatal health conditions, and VPDs in migrant populations in the MENA region.”<br><br>What would be the difference between prevalence of a disease and morbidity (e.g. between prevalence of depression and morbidity related to depression)? And would not intervention coverage be an indicator of service use rather than an indicator of disease? These terms and indicators need to be clarified.	We thank you for flagging this lack of clarity. We have discussed the outcomes and are happy to re-categorise as follows (which is also reflected in our response to question 6 and question 12, pages 6-7, 10, 12-15):<br><br>- Burden outcomes (e.g., prevalence or incidence)<br>- Invention outcomes (e.g., uptake and coverage of screening or treatment, treatment success)<br>- Intermediate outcomes (e.g., severity of disease, prognosis)<br>- Final outcomes (e.g., mortality, quality of life)
16	Would not c be an integrated part of objective a and b?	We wanted to make it explicit that we will be synthesising the evidence AND appraising the quality of evidence.<br><br>We have now incorporated the word appraise into the remaining objectives and dropped the separate objective on quality appraisal (see response to question 12 and pages 6-7).
17	Research question 2, form the methods:<br>“For the policy-related data (question 2), we will include papers that contain	We thank you for flagging this point. This is true, we will be including studies on barriers and facilitators of health services related to the diseases areas that are not included in

No	Comment	Response
	a description of the policies themselves, uptake of the health services mentioned in the policies and determinants of any under-usage, and facilitators or barriers in accessing the health services mentioned in the policies for the diseases in migrant populations in the MENA region. Definitions for migrant and the MENA region are described in panel 1."<br><br>What about use of regular health services and barriers to that? Most health services will probably be regular services not described in policies regarding migrants especially?	policies regarding migrants. We have split the two questions up, adding a third question about the barriers and facilitators, as follows (this is also reflected in our response to question 6 and 12, pages 6-7, 10,12-15):<br><br>2. *What is the policy response for each disease area related to migrant populations in the MENA region?*<br>*Objective:*<br>a. *Synthesise and appraise the prevention and/or treatment policies for TB, HIV, hepatitis B and C, malaria, NTDs, diabetes, mental health, maternal and neonatal health, and VPDs in migrant populations in the MENA region.*<br><br>3. *What are the barriers and facilitators in accessing health services for each disease area for migrant populations in the MENA region?*<br>*Objective:*<br>a. *Synthesise and appraise the evidence on the barriers and facilitators for accessing prevention and/or treatment services for TB, HIV, hepatitis B and C, malaria, NTDs, diabetes, mental health, maternal and neonatal health, and VPDs in migrant populations in the MENA region.*
18	Search strategies: "An iterative procedure was used, with input from all authors including an information scientist, recommended search filters, and previous reviews."<br><br>Can you explain what input from recommended search filters could be?	We looked at the following sites to search for relevant search terms to inform our strategy. We have now referenced them in the manuscript (page 8).<br><br>- https://hiruweb.mcmaster.ca/hkr/hedges/medline/<br>- https://sites.google.com/a/york.ac.uk/issg-search-filters-resource/home/recently-added-filters<br>- https://cks.nice.org.uk/topics/hiv-infection-aids/how-this-topic-was-developed/search-strategy/<br>- https://cks.nice.org.uk/topics/malaria/how-this-topic-was-developed/search-strategy/<br>- https://cks.nice.org.uk/topics/tuberculosis/how-this-topic-was-developed/search-strategy/

No	Comment	Response
19	It can be considered to start the search even later than 2000, as almost 25 years will have passed when the results are ready, and earlier information may not be especially relevant anymore.	We agree that the earlier results may not be as relevant anymore, however, we have searched up to 2000 to see if there are any trends over time. Year of data collection is something we will consider when synthesising the results. We have added “study period” in the methods to show this (pages 7 and 17-18):<br><br>*“We will disaggregate by, and investigate potential sources of heterogeneity for, country of study, study period, country of origin, migrant type (i.e., labour, asylum seekers, refugee), age, and sex where feasible for all objectives, and cross-compare findings across countries in the MENA region.”* (page 7)<br><br>*“If there are sufficient data, we will investigate potential sources of heterogeneity, using meta-regression, and incorporating the following covariates in each model: country of study, study period, type of migrant (labour, asylum seeker, refugee, undocumented, etc.); setting/housing (camps, community, detention etc.); comorbidities; country of birth/origin; age and sex.”* (page 17, for meta-analysis for question 1 disease indicators)<br><br>*“We will stratify the results by outcome and type of migrant and investigate heterogeneity qualitatively by exploring differences in results by country of study, study period, setting/housing, country of birth/origin, etc., as appropriate.”* (page 18, for question 1 where meta-analysis is not possible)<br><br>*“We will also stratify these results by type of migrant, country of study, study period, setting/housing, and country of birth/origin, etc., as appropriate.”* (page 18, for questions 2 and 3)
20	You have no language restriction: What is the plan is you come across a study in a language none of the authors have competencies in?	Amongst the colleagues in our consortium, we have speakers that cover the main languages of the Middle East and North Africa, including Arabic, French and English. On the rare chance that we find an article in another language, we will use a professional

No	Comment	Response
		translator or an online automated translation service, depending on the costs and budgets. We have added the following sentence about this in the methods on page 11:<br><br>*"We will have no language exclusions as within our co-authors we have speakers of the MENA region (Arabic, French and English) as well as Spanish; if we find an article in another language, we will use a professional translator or an online automated translation service, depending on the cost and budget."*
21	Why have you chosen diabetes and not other NCDs? You have included very many different VPDs, so why not include other NCDs like hypertension, CVDs, stroke or cancer? This would have been just as relevant, thus this choice needs an explanation. And what about including hyperglycemia as a search term with diabetes?	As mentioned in our response to question 3, we wanted to cast a wide net across disease areas that might be important to the different migrant groups, thus we have tried to cover as many key communicable and non-communicable diseases as possible. We acknowledge that for non-communicable diseases we have only included maternal and neonatal health conditions, mental health, and diabetes. We initially planned to look at multiple non-communicable diseases, however, the number of hits was unmanageable, so we chose all maternal and neonatal health conditions, all mental health conditions, and diabetes. We chose diabetes as, in our scoping for this review, we found diabetes to be the disease of most concern for migrants in the region (which we reference in the introduction).<br><br>We do agree it is important to look at other conditions so we are pleased to inform you that while we will include diabetes at this first stage, in the next stage we will do a systematic review on other NCDs, including hypertension, CVD, obesity and CKD. We have added a sentence about this choice and the next steps in the methods (page 11) and in a limitations section at the end of the methods acknowledging this and other limitations (pages 18-19):

No	Comment	Response
		“.. In the next stage we will do a wider systematic review on NCDs in migrant populations in the MENA region, including hypertension, cardiovascular diseases, obesity, and chronic kidney disease..” (page 11)<br><br>*“Another limitation in the scope of the NCDs systematic review is that we are limiting the diseases area to diabetes only. This is to make the suite of reviews feasible, however, it is not representative of the literature on all NCDs. Once this suite of reviews is completed, we will undertake a second review on NCDs in migrant populations in the MENA region, including hypertension, cardiovascular disease, obesity, and chronic kidney disease.”* (pages 18-19)<br><br>As for the term hyperglycaemia, we will be including it and exploring any additional papers that are identified.
22	For mental health: You have included some more general search terms like “mental health” and mental disorders” and some common mental disorders like “anxiety” and depression”. You have also a lot of diagnoses which is not common/correct to include as a mental disorder, like “diffuse neurofibrillary tangles with calcification”, “relative energy deficiency in sport”, “vaginisimus” and erectile dysfunction”, and others which seems of little relevance, like “kinesiophobia*. Other terms which I would consider much more relevant, such as “post traumatic stress syndrome” is not included. Moreover, various substance use disorders are included as mental health (which it is not, although they might co-appear) and also tobacco use. I would suggest to revise included search terms and eliminate all those not relevant, and also to include an own search for substance use disorders.	The definition we used for mental health and the inclusion of search terms comes directly from the medical subject headings (MeSH) and thesaurus of Medline / PubMed. This includes terms like “diffuse neurofibrillary tangles with calcification”, “relative energy deficiency in sport”, “vaginisimus”, “erectile dysfunction”, “kinesiophobia*, and various substance use disorders and tobacco use.<br><br>Therefore, we would argue that these conditions are relevant and, as mentioned above, we are trying to cast a wide net. Furthermore, including them simply means that we would pick such articles up and can then decide how to synthesise them at the next stage (i.e., perhaps separating them into different types of mental health disorders). It does not have any major implications to the review findings.

No	Comment	Response
		There is a misunderstanding that post-traumatic stress disorders are not included. Post-traumatic stress disorders are included in our search strategy as follows:<br><br>1) In free text search: trauma adj3 disorder*.ti,ab,kf.<br>2) In the use of MeSH terms: They are included in the exploding of the search term 'mental disorders', which includes:<br>- Trauma and Stressor Related Disorders<br>- Adjustment Disorders<br>- Stress Disorders, Traumatic<br>- Battered Child Syndrome<br>- Combat Disorders<br>- Psychological Trauma<br>- Sexual Trauma<br>- Stress Disorders, Post-Traumatic<br>- Stress Disorders, Traumatic, Acute
23	In general; the choice of search terms seems a bit random, and would benefit from better clarification and arguments.	As above, this might be a misunderstanding. The choice of search terms is not random, and much thought and input has gone into this process. It has been informed by an information scientist, topic experts, and all authors, which then led to the final agreed strategies.
24	Table 2: same comment as above, it is not clear what is really the difference between "burden" and "final clinical outcome" and "intermediate clinical outcome". This needs to be explained (and maybe re-named?)	We thank you again for flagging this and have made the changes to the table (pages 12-15) and throughout the manuscript to edit the outcomes as follows (in line with our responses to questions 6 ,12, 15, and 17):<br><br>- Burden outcomes (e.g., prevalence or incidence)<br>- Invention outcomes (e.g., uptake and coverage of screening or treatment, treatment success)<br>- Intermediate outcomes (e.g., severity of disease, prognosis)<br>- Final outcomes (e.g., mortality, quality of life)
25	Should the flow chart been modified to better reflect the process of including grey literature and the extensive search in policy documents?	This is the standard template of the flowchart recommended by PRISMA. In this diagram, grey literature would come into the box on "Additional records identified through other sources".<br><br>Depending on what we find in the various sources and how many sources we end up with, we will place the sources and number of articles within this box, or we will make a

No	Comment	Response
		separate table for grey literature sources if this more presentable. We have added the following footnote to explain this:<br><br>*“Note: We will edit this flow diagram as appropriate to clearly display the grey literature sources we find.”*
26	Ethics and dissemination:<br>“We also intend to report the findings to ministries of health in Morocco, Tunisia, and Sudan<br>where we will be conducting the qualitative studies to continue the development of the MHCP-t.” The qualitative studies referred to have not been described in the article?	We mentioned in the introduction that these systematic reviews are a preliminary step in the development of the MHCP-t. The qualitative studies are part of the other steps in the process of developing the MHCP-t. We have now briefly mentioned the following additional steps of the qualitative studies in the introduction section on page 5 and referred to this in the ethics and dissemination section:<br><br>*“We will also conduct qualitative field studies with migrants, community leaders, and healthcare professionals in Morocco, Tunisia, and Egypt to further inform the key indicators. The resulting key indicators will be reviewed by national task groups, brought together by the ministries of health, and international experts. The final list of indicators will be developed into the first version of the tool, which will be piloted within the countries using a mixed methods process evaluation.”*
